# Elimination of TDP-43 inclusions linked to amyotrophic lateral sclerosis by a misfolding-specific intrabody with dual proteolytic signals

**DOI:** 10.1038/s41598-018-24463-3

**Published:** 2018-04-16

**Authors:** Yoshitaka Tamaki, Akemi Shodai, Toshifumi Morimura, Ryota Hikiami, Sumio Minamiyama, Takashi Ayaki, Ikuo Tooyama, Yoshiaki Furukawa, Ryosuke Takahashi, Makoto Urushitani

**Affiliations:** 1 Department of Neurology, Shiga University of Medical Science, Otsu, Japan; 2 Department of Neurology, Kyoto University Graduate School of Medicine, Kyoto, Japan; 3 Molecular Neuroscience Research Center, Shiga University of Medical Science, Otsu, Japan; 4 Department of Chemistry, Keio University, Yokohama, Japan

## Abstract

Aggregation of TAR DNA-binding protein of 43 kDa (TDP-43) is implicated in the pathogenesis of sporadic and certain familial forms of amyotrophic lateral sclerosis (ALS), suggesting elimination of TDP-43 aggregates as a possible therapeutic strategy. Here we generated and investigated a single-chain variable fragment (scFv) derived from the 3B12A monoclonal antibody (MAb) that recognises D247 of the TDP-43 nuclear export signal, an epitope masked in the physiological state. In transfected HEK293A cells, 3B12A scFv recapitulated the affinity of the full-length MAb to mislocalised TDP-43 with a defective nuclear localising signal and to a TDP-43 inclusion mimic with cysteine-to-serine substitution at RRM1. Moreover, 3B12A scFv accelerated proteasome-mediated degradation of aggregated TDP-43, likely due to an endogenous PEST-like proteolytic signal sequence in the VH domain CDR2 region. Addition of the chaperone-mediated autophagy (CMA)-related signal to 3B12A scFv induced HSP70 transcription, further enhancing TDP-43 aggregate clearance and cell viability. The 3B12A scFv also reduced TDP-43 aggregates in embryonic mouse brain following *in utero* electroporation while causing no overt postnatal brain pathology or developmental anomalies. These results suggest that a misfolding-specific intrabody prone to synergistic proteolysis by proteasomal and autophagic pathways is a promising strategy for mitigation of TDP-43 proteinopathy in ALS.

## Introduction

Amyotrophic lateral sclerosis (ALS) is a devastating neurodegenerative disease characterised by progressive muscle wasting and weakness, leading ultimately to lethal respiratory failure. Multiple pathogenic pathways have been implicated in the motor neuron degeneration of ALS, such as excitotoxicity, neuroinflammation, proteolysis impairment, oligodendrocyte dysfunction, mitochondrial dysfunction and disruption of RNA homeostasis^1–4^. Emerging evidence suggests that the accumulation of various disease-related misfolded proteins may underlie these pathogenic processes. Despite advances in understanding ALS pathogenesis, however, there is no curative or effective control therapy.

The TAR DNA-binding protein of 43 kDa (TDP-43) is a DNA/RNA-binding protein predominantly located in the nucleus under physiological conditions. However, hyperphosphorylated, fragmented and ubiquitinated forms of TDP-43 were identified as core components of cytosolic inclusions in sporadic ALS and frontotemporal lobar degeneration (FTLD)^5–9^. TDP-43 contains a nuclear localising signal (NLS) as well as a nuclear export signal (NES)^10^, which enables the physiological shuttling of TDP-43 between nucleus and cytosol. Under stress-free conditions, TDP-43 interacts with mRNAs on which ribosomes are located separately, forming polysomes. Various stresses induce clustering of ribosomes into a ‘stalled’ state, resulting in the formation of stress granules (SG) containing TIA-1, G3BP, ataxin-2 and eIF4G1/2. In the stalled state, transcription is inhibited as a homeostatic response. However, sustained stress and ensuing TDP-43 misfolding creates aberrant SGs and pathogenic TDP-43 aggregates^4,11,12^. Moreover, membrane-less organelles in the cytosol formed by the liquid−liquid phase separation of RNPs^4^ and RNA^13^ are implicated in TDP-43 proteinopathy. Misfolding and cytosolic mislocalisation also lead directly to a loss of normal TDP-43 function, and the resultant disruption of protein and RNA homeostasis is considered another likely pathogenic mechanism^14^ in addition to the toxicity of inclusions. TDP-43 inclusions can be identified in the majority of ALS patients with the exception of patients with superoxide dismutase 1 (SOD1) mutations^7,14,15^, suggesting that these aggregates are a core pathology of sporadic ALS. Aberrant TDP-43 inclusions are also observed in familial ALS (FALS) cases associated with various mutations such as *C9orf72*^16^, *MATR3*^17^, *hnRNP1*^18^, *UBQLN2*^19^, *SQSTM1*^20^, *VCP*^21^ and *OPTN*^22^, as well as in cases with mutations of the TDP-43 gene (*TARDBP*) itself, indicating that misfolded TDP-43 is a common pathogenic molecule in familial as well as sporadic ALS. These lines of evidence suggest that targeting pathogenic TDP-43 species is a possible therapeutic strategy against ALS.

A transgenic mouse model with doxycycline (DOX)-dependent suppression of mutant human TDP-43 harbouring a defective nuclear localising signal (NLS) exhibited accumulation of cytoplasmic TDP-43 aggregates, loss of endogenous nuclear TDP-43 and progressive motor impairments in the DOX-OFF state^23^. Unexpectedly, the suppression of cytoplasmic human TDP-43 expression after disease onset (DOX-ON) reversed TDP-43 pathology and rescued the motor deficits^23^. These results indicate that mislocalised TDP-43 is directly toxic and further suggest that removal of misfolded TDP-43 is a promising therapeutic strategy for TDP-43-linked ALS, even after formation of TDP-43 inclusions. Ubiquitination and phosphorylation of TDP-43 are pathological hallmarks of sporadic ALS. However, they are relatively late phenomena in ALS progression^24–26^, and thus targeting the early conformational changes in pathogenic TDP-43 is required for pre-emptive therapy. We previously identified E246 and D247 as molecular epitopes of the mislocalised/misfolded TDP-43^27^. These residues are located at the assembly interface of the RNA Recognition Motif 2 (RRM2) domain formed upon interaction with single-stranded DNA^28^. Using these residues as epitopes, we successfully generated a misfolding-specific monoclonal antibody (MAb), 3B12A, which recognises mislocalised or misfolded TDP-43 in cultured cells and spinal cord sections from ALS patients, but not wild-type (WT) TDP-43 in the nucleus^27^.

Here we report the development of self-degradative intrabodies derived from 3B12A MAb, and demonstrate effective elimination of misfolded TDP-43 both *in vitro* and *in vivo*. This effect was mediated by dual proteolytic signals, an endogenous PEST-like signal and a chaperone-mediated autophagy (CMA)-localising signal. Moreover, aggregate binding to CMA-fused 3B12A scFv unexpectedly induced heat shock protein (HSP) 70, which further enhanced TDP-43 clearance by promoting protein refolding.

## Results

### Generation of 3B12A intrabodies targeting misfolded TDP-43

**Figure 1:**
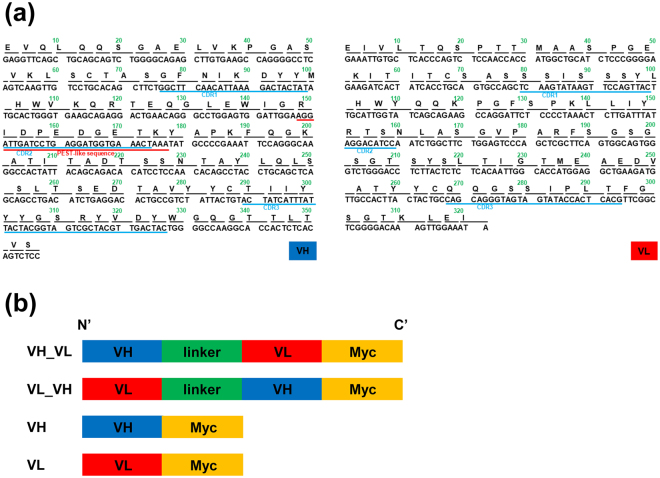
The construction of 3B12A intrabodies against misfolded TDP-43. (**a**) DNA and peptide sequences of VH (left panel) and VL (right panel). VH contains residues with high PEST score, thus referred to as a PEST-like sequence, in the CDR2 domain. (**b**) Domain profiles of 3B12A intrabodies. After cloning cDNAs encoding variable fragments of the heavy chain (VH) and light chain (VL) from a hybridoma expressing 3B12A IgG, we constructed plasmids encoding single-chain variable fragments (scFv) as 3B12A intrabodies with Myc-tag.

We first aimed to develop 3B12A MAb-derived intrabodies targeting only the pathogenic structure of TDP-43. Previous reports demonstrated that the 3B12A MAb selectively reacts with aggregated or cytosolic mislocalised TDP-43 in cultured cells and postmortem spinal cord from sporadic ALS patients^27,29^. To apply this antibody inside cells as a potential scavenger of intracellular TDP-43 aggregates, we generated a single-chain variable fragment (scFv) to be used as an intrabody. The cDNAs encoding variable fragments of the heavy chain (VH) and light chain (VL) were obtained from hybridoma 3B12A mRNA, and domain profiles were investigated. Unexpectedly, DNA sequence analysis of these 3B12A intrabodies showed that the VH domain contains a RIDPEDGETK sequence with PEST score as high as 9.02 at the complementarity determining region 2 (CDR2; Fig. 1a), potentially conferring the capacity to induce proteasome-mediated proteolysis^30–32^. Four 3B12A-derived intrabodies were generated: VH-linker-VL (VH_VL) and VL-linker-VH (VL_VH) as scFv forms and VH and VL as nanobodies tagged with Myc at the C-terminus (Fig. 1b).

### 3B12A intrabodies interact with misfolded TDP-43 inside cells

**Figure 2:**
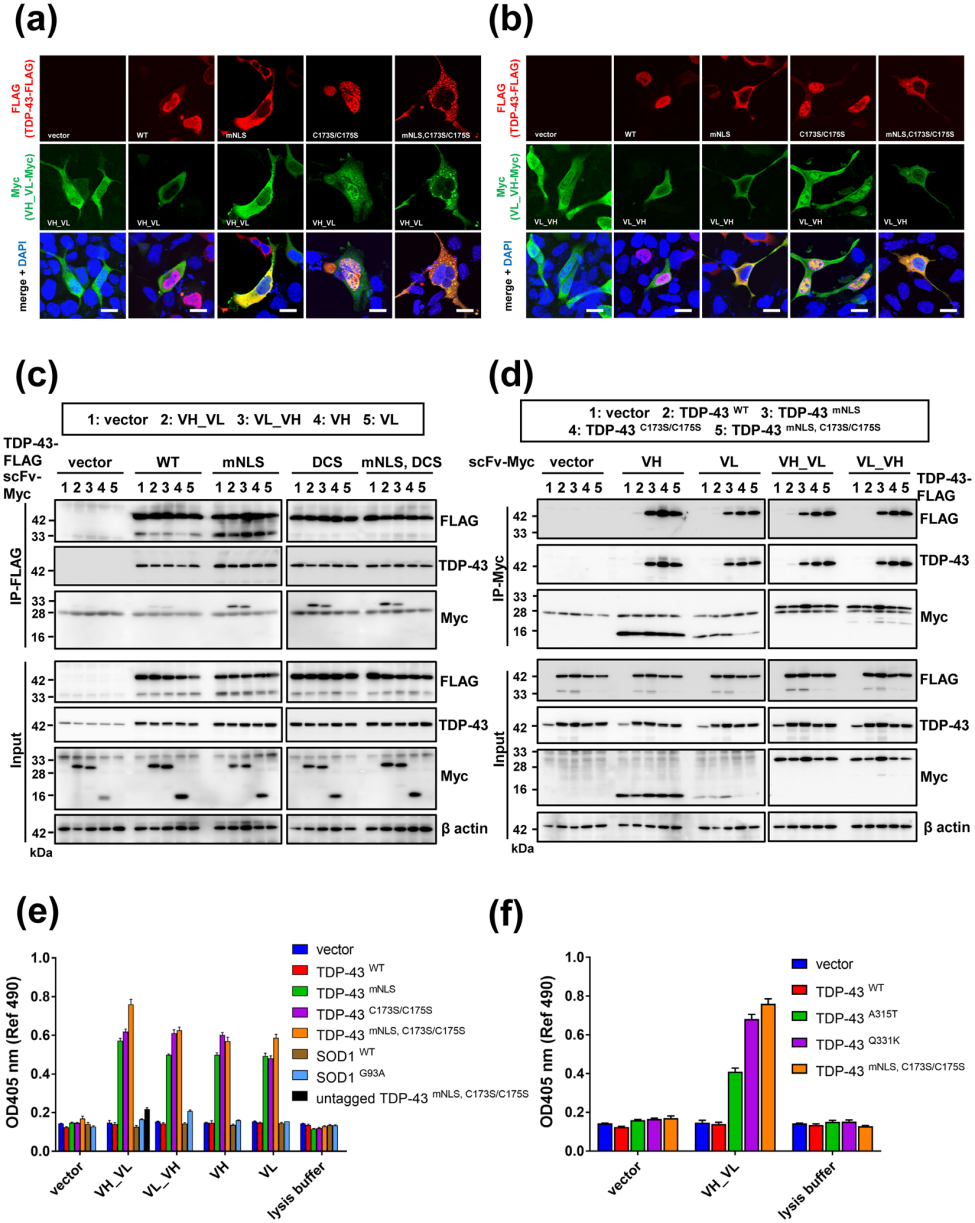
3B12A intrabodies selectively interact with misfolded and/or mislocalised TDP-43. (**a**,**b**) Confocal laser micrographs of HEK293A cells expressing TDP-43-FLAG (red) and Myc-tagged 3B12A intrabodies (green) at 48 h after transfection. DAPI was used for counterstaining of nuclei (blue). Scale bar = 20 µm. (**c**) 3B12A scFv (VH_VL or VL_VH) interacted with cytoplasmic TDP-43 (TDP-43^mNLS^) and aggregated TDP-43 (TDP-43^C173S/C175S^ and TDP-43^mNLS,C173S/C175S^). DCS indicates the C173S/S175S mutant. (**d**) TDP-43^C173S/C175S^, TDP-43^mNLS,C173S/C175S^ and TDP-43^mNLS^ were also immunoprecipitated with both 3B12A scFv and 3B12A nanobodies (VH or VL). (**e**) Sandwich ELISAs showing the specific immunoreactivity of 3B12A intrabodies against TDP-43 species. Lysates from HEK293A cells co-transfected with Myc-tagged 3B12A intrabodies and FLAG-tagged TDP-43 or SOD1 (WT or G93A mutant) were reacted to ELISA plates previously coated with anti-FLAG antibody and subsequently detected with anti-Myc antibody. Untagged-TDP-43^mNLS, C173S/C175S^ was used as a negative control. (**f**) Sandwich ELISA showing the immunoreactivity of 3B12A scFv for the familial ALS-linked TDP-43 mutants A315T or Q331K.

We investigated the antigen specificity of these 3B12A-derived intrabodies by immunocytochemistry, immunoprecipitation assays and sandwich ELISA using co-transfected HEK293A cells. Our previous study revealed that the conformation of TDP-43 is regulated by two cysteine residues in RRM1, and that substitution to serine (C173S and/or C175S) leads to the formation of TDP-43 aggregates sharing pathological and functional features of the inclusions observed in sporadic ALS^33^. We thus used the C173S/C175S mutant (TDP-43^C173S/C175S^) as a model for misfolded and aggregated TDP-43, and TDP-43 with an NLS mutation (TDP-43^mNLS^)^34^ as a TDP-43 cytosolic aggregate mimic in the present study (Supplementary Fig. S1). Confocal laser microscope analysis of HEK293A cultures overexpressing a FLAG-tagged TDP-43 (WT or mutant) and one of the four 3B12A intrabodies showed that Myc-tagged VH_VL colocalised with TDP-43^mNLS^, TDP-43^C173S/C175S^ and aggreagated cytoplasmic TDP-43 with both mutations (TDP-43^mNLS,C173S/C175S^), but not with TDP-43^WT^. Moreover, VH_VL preferentially colocalised with TDP-43 aggregates over TDP-43^WT^ whether in the nucleus or cytosol (Fig. 2a). Likewise, Myc-tagged VL_VH colocalised with cytosolic TDP-43 and TDP-43 aggregates (Fig. 2b). These results suggest that both 3B12A scFv forms preferentially recognise misfolded and mislocalised TDP-43 inside cells regardless of subcellular distribution, while neither binds normally folded TDP-43. Both nanobodies also preferentially bound cytosolic TDP-43 and aggregated mutant TDP-43, although less extensively than VH_VL scFv (Supplementary Fig. S2). These 3B12A intrabodies also partially colocalised with SOD1^G93A^ inclusions (Supplementary Fig. S3), possibly due to a HSP70-mediated or nonspecific interaction.

Subsequent immunoprecipitation assays of HEK293A cells co-overexpressing a Myc-tagged 3B12A intrabody and a FLAG-tagged TDP-43 showed that both VH_VL and VL_VH preferentially pulled down TDP-43^mNLS^, TDP-43^C173S/C175S^ and TDP-43^mNLS,C173S/C175S^ over TDP-43^WT^ (Fig. 2c). Reverse immunoprecipitation using Myc antibody yielded consistent results, with TDP-43^mNLS^, TDP-43^C173S/C175S^ and TDP-43^mNLS,C173S/C175S^ mutants interacting with both 3B12A scFv forms and 3B12A nanobodies, whereas TDP-43^WT^ did not (Fig. 2d).

We next compared the specific reactivities of 3B12A intrabodies to TDP-43 species using sandwich ELISA assay. Lysates from cultured HEK293A cells co-transfected with Myc-tagged 3B12A intrabodies and FLAG-tagged TDP-43 or SOD1 species were incubated on ELISA plates previously coated with anti-FLAG antibody and binding subsequently detected with anti-Myc antibody (Fig. 2e,f) or anti-TDP-43 antibody (Supplementary Fig. S4a). Quantitative analysis by sandwich ELISA showed the preferential interaction of VH_VL scFv with the cytoplasmic aggregation-prone TDP-43 mutant TDP-43^mNLS,C173S/C175S^ compared with VL_VH, VH and VL, as well as the lower affinity to TDP-43^WT^ and SOD1 species including SOD1^G93A^ mutant (Fig. 2e). The VH_VL intrabody also showed higher affinity to familial ALS-linked A315T and Q331K mutants of TDP-43 than to TDP-43^WT^ (Fig. 2f) although affinity was lower than to the C173S/C175S mutant. Moreover, both 3B12A scFv forms showed a higher affinity to TDP-43^mNLS,C173S/C175S^ than C4F6 scFv, an intrabody derived from C4F6 MAb^35^ raised against misfolded SOD1 mutant SOD1^G93A^ (Supplementary Fig. S4b). Thus, all four 3B12A intrabodies demonstrated high affinity for misfolded TDP-43, particulally VH_VL scFv, although reactivity to the native form varied. Furthermore, VH_VL scFv also showed a higher reactivity to other ALS-linked TDP-43 mutants than to WT. However, the discrepancy between sandwich ELISA and immunoprecipitation experiments regarding aggregate binding of VH or VL nanobodies implies that a nonspecific interaction, possibly based on the insolubility of nanobodies, may be responsible for the interactions of these forms with aggregated TDP-43 observed in ELISA. Based on these results, we used VH_VL scFv for subsequent examination of TDP-43 clearance.

### Chaperone-mediated autophagy signal confers bi-directional proteolytic properties to 3B12A VH_VL scFv

**Figure 3:**
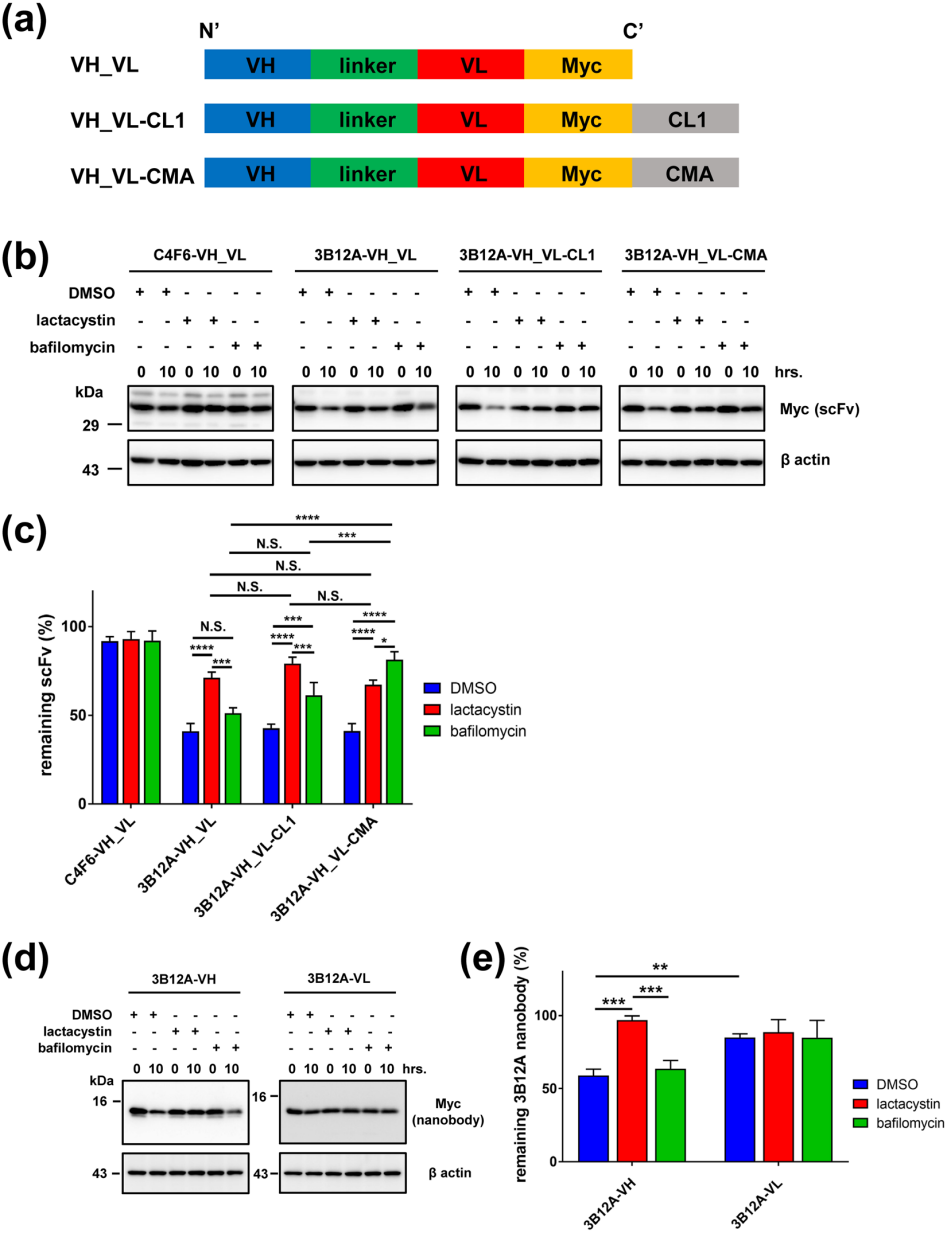
3B12A scFv-CMA is degraded by both proteasome and autophagy proteolytic pathways. (**a**) Illustrative domain profiles of 3B12A scFv for VH_VL-Myc with or without proteolytic signals including CL1 for proteasomes and chaperone-mediated autophagy (CMA) for autophagosomes. (**b**) Protein degradation chase assay of C4F6 scFv (C4F6-VH_VL), 3B12A scFv (VH_VL), 3B12A scFv-CL1 (VH_VL-CL1) and 3B12A scFv-CMA (VH_VL-CMA) in HEK293A cells. (**c**) Quantitative analysis of (**b**) Each data point was obtained by normalisation to actin. Differences were evaluated by one-way ANOVA (mean ± SD from three independent experiments; *p < 0.05, ***p < 0.005 and ****p < 0.001). N.S. indicates not significant. ‘remaining scFv (%)’ indicates ‘the % signal compared with time 0’. (**d**) Protein degradation chase assay of 3B12A-VH and 3B12A-VL in HEK293A cells. (**e**) Quantified analysis of (**d**) Each data point was obtained by normalisation to actin. Differences were evaluated by one-way ANOVA (mean ± SD from three independent experiments; **p < 0.01 and ***p < 0.005).

Self-degradation is a desirable property for a therapeutic intrabody, so we investigated the effects of the VH domain PEST-like sequence and protein disassembling signals on the degradation of 3B12A VH_VL scFv via proteasomal and autophagic–lysosomal proteolysis pathways using a cycloheximide (CHX) chase study. For this purpose, we constructed a VH_VL intrabody containing two protein disassembling signals, CL1 for proteasome localisation^36,37^ and CMA for chaperone-mediated autophagy^38,39^ (Fig. 3a), based on the assumption that the interaction between scFv and antigen alone may not suffice for protein clearance. The chase assay revealed that 3B12A scFv (with a PEST-like sequence) declined more rapidly than C4F6 scFv without a PEST-like sequence, and proteasome inhibition by lactacystin prevented this decline (Fig. 3b,c). The intracellular concentration of modified 3B12A scFv containing the CL1 signal (VH_VL-CL1) also declined rapidly over 10 h following CHX application, and this decline was prevented by lactacystin (Fig. 3b,c). Furthermore, VH_VL-CL1 detection by Western blot analysis required a longer ECL exposure time than required for VH_VL alone, suggesting that the CL1 signal accelerated VH_VL degradation in proteasomes. The intracellular level of 3B12A scFv containing a CMA signal (VH_VL-CMA) declined as rapidly as VH_VL and VH_VL-CL1, and this decrease was inhibited by the lysosome inhibitor bafilomycin as well as by lactacystin (Fig. 3b,c). Consistent with lysosomal degradation, the effect of bafilomycin was significantly greater on CMA-fused VH_VL than VH_VL-CL1 or VH_VL alone. Lactacystin significantly reversed the degradation of all 3B12A scFv types. The lack of a significant difference in elimination rate among these different VH_VL forms may be due to the endogenous PEST-like signal. Indeed, less of the VH fragment (containing the PEST-like sequence) remain at 10 h after the chase compared with the VL fragment, and lactacystin significantly inhibited VH degradation. This finding indicates that the VH domain PEST-like sequence regulates its turnover (Fig. 3d,e). We confirmed that neither CHX, lactacystin, nor bafilomycin was cytotoxic, further indicating that intrabody decline resulted from specific proteasome- and lysosome-mediated degradation (Supplementary Fig. S5).

We also confirmed that proteasome activity was not suppressed by 3B12A scFv alone (Supplementary Fig. S6a), TDP-43^WT^ or misfolded TDP-43 species (Supplementary Fig. S6b) in the presence and absence of 3B12A scFv (Supplementary Fig. S6c). Likewise, measurement of LC3-II/LC3-I flux by Western blot analysis showed that autophagy–lysosomal activity was not altered by intrabodies containing protein disassembling signals in the presence and absence of WT or misfolded TDP-43 (Supplementary Fig. S6d,e).

### 3B12A scFv-CMA facilitates the degradation and elimination of misfolded TDP-43 in the cytosol

**Figure 4:**
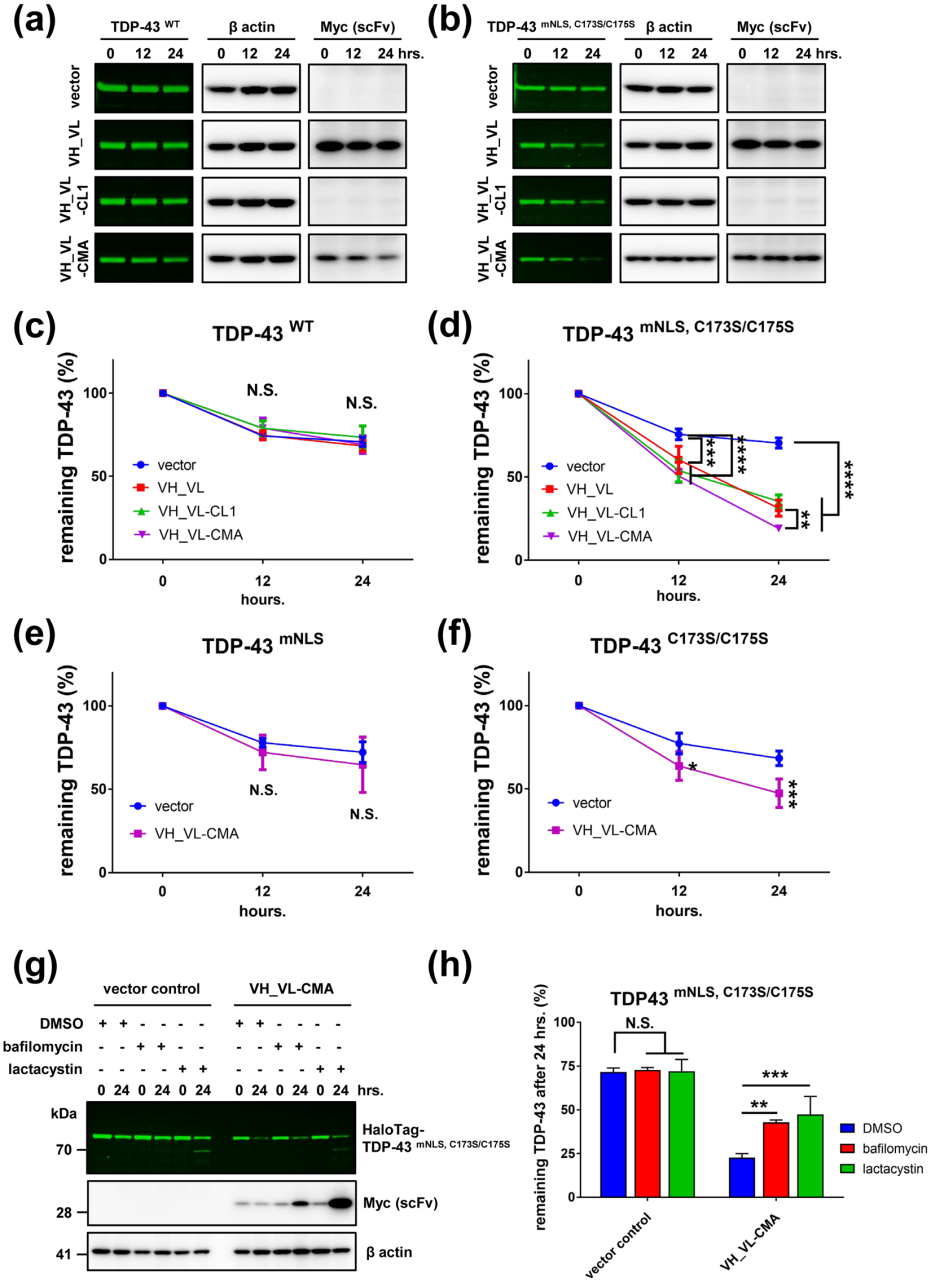
3B12A scFv selectively degrades aggregated TDP-43 and the CMA signal further promotes degradation of aggregated TDP-43. (**a**,**b**) HaloTag pulse chase assay of TDP-43^WT^ and TDP-43^mNLS,C173S/C175S^. (**c**,**d**) Quantified data for HaloTag pulse chase assay of (**a**,**b**) respectively. Each data point was obtained by normalisation to actin. 3B12A scFvs promoted the degradation of the TDP-43^mNLS,C173S/C175S^ mutant. Moreover, VH_VL-CMA significantly enhanced degradation compared with VH_VL. Differences were evaluated by two-way ANOVA (mean ± SD from three independent experiments; **p < 0.01, ***p < 0.005 and ****p < 0.001). N.S. indicates not significant. (**e**,**f**) Quantified data for HaloTag pulse chase assay of TDP-43^mNLS^ and TDP-43^C173S/C175S^, respectively. Differences were evaluated by two-way ANOVA (mean ± SD from three independent experiments; *p < 0.05 and ***p < 0.005). N.S. indicates not significant. (**g**) HaloTag pulse chase assay for cytoplasmic aggregated TDP-43 in the presence of lactacystin or bafilomycin. (**h**) Quantitative analysis of (**g**) Each data point was obtained by normalisation to actin. Differences were evaluated by one-way ANOVA (mean ± SD from three independent experiments; **p < 0.01 and ***p < 0.005 *vs* DMSO control).

To evaluate the efficacy of the 3B12A intrabody for intracellular clearance of misfolded TDP-43, we performed HaloTag pulse chase assays in which a covalent bond between a HaloTag-fused protein and synthetic ligands enables the transient labelling of target proteins in live cells. HEK293A cells overexpressing HaloTag-fused TDP-43 with or without VH_VL 3B12A scFv intrabody co-expression were labelled with a cell membrane-permeable HaloTag fluorescent ligand (HaloTag diAcFAM ligand), and cell lysates were analysed at several time points post-labelling. The HaloTag pulse chase assay demonstrated that 3B12A scFv co-expression markedly enhanced the degradation of the cytoplasmic aggregation-prone TDP-43 mutant TDP-43^mNLS,C173S/C175S^ at 12 and 24 h after adding HaloTag diAcFAM ligand, whereas 3B12A scFv did not promote clearance of TDP-43^WT^ (Fig. 4a–d). Moreover, the addition of a CMA signal (VH_VL-CMA) accelerated the degradation of aggregated TDP-43 compared with VH_VL 3B12A scFv or VH_VL-CL1 (Fig. 4b,d). Co-expression of VH_VL-CMA also significantly enhanced the degradation of the nuclear aggregated TDP-43 mutant (TDP-43^C173S/C175S^) but did not significantly accelerate clearance of the cytoplasmic TDP-43 mutant (TDP-43^mNLS^) (Fig. 4e,f), confirming that VH_VL-CMA has higher affinity to misfolded species. Furthermore, this effect was inhibited by either lactacystin or bafilomycin (Fig. 4g,h), indicating that cytoplasmic TDP-43 aggregates are eliminated by VH_VL-CMA through both the proteasomal and autophagy–lysosome pathways.

### 3B12A scFv-CMA eliminates cytoplasmic TDP-43 inclusions and mitigates the cytotoxicity induced by misfolded TDP-43

**Figure 5:**
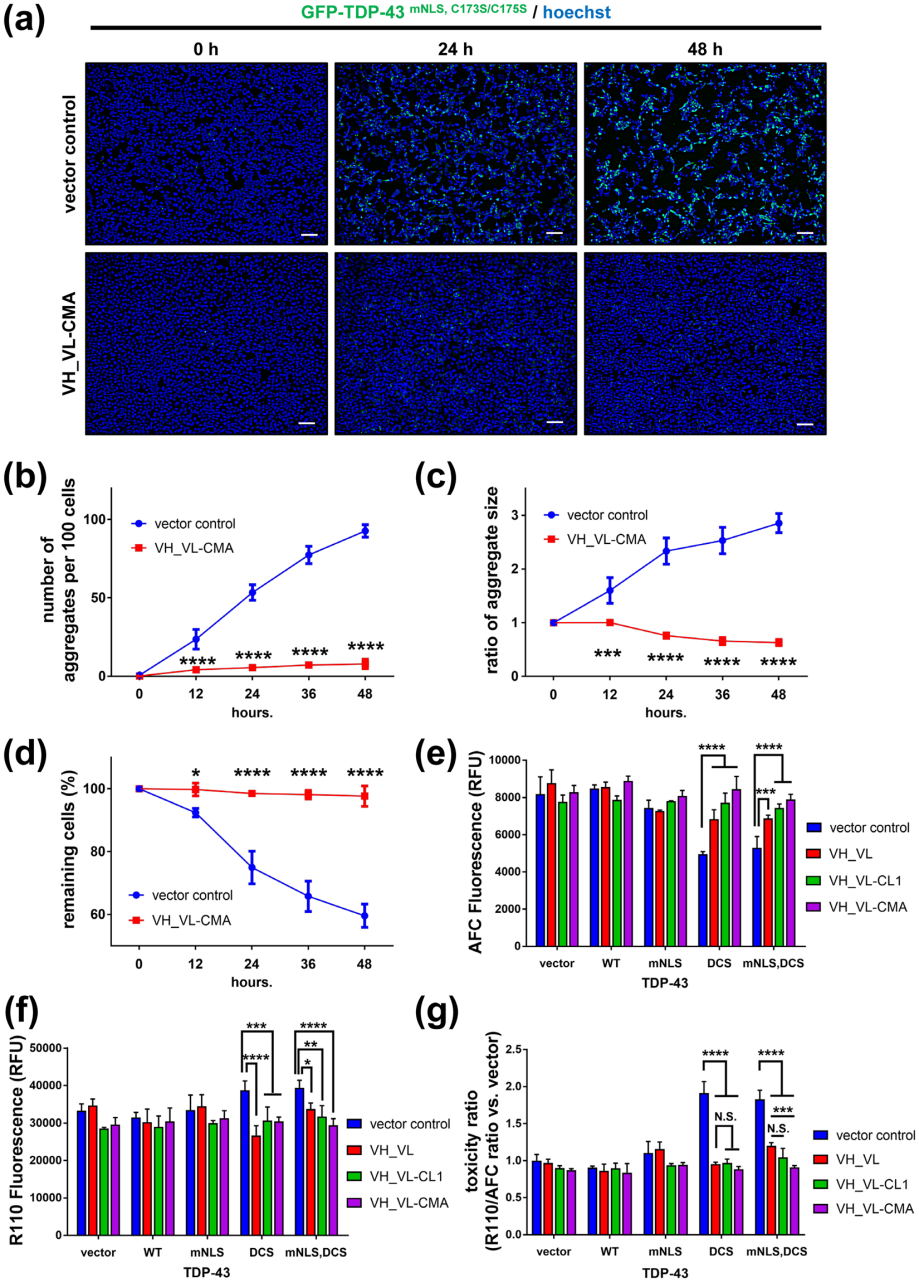
3B12A scFv prevents the growth and proliferation of cytoplasmic TDP-43 aggregates to reduce cell death. (**a**) Time-lapse imaging of cytoplasmic TDP-43 aggregates in the presence or absence of 3B12A scFv-CMA in HEK293A cells. Scale bar = 100 µm. (**b–d**) Quantified analysis of time-lapse imaging. TDP-43 aggregates were distinguished from non-aggregates by diminishing the fluorescence intensity of diffusely distributed cytoplasmic GFP-TDP-43 signals and subsequently quantified with ImageJ software. Differences were evaluated by two-way ANOVA (mean ± SD from three independent visual fields; *p < 0.05, ***p < 0.005 and ****p < 0.001). (**e**,**f**) Effects of TDP-43 species and 3B12A scFv on N2a cell toxicity (**e**) and viability (**f**) DCS represents the C173S/C175S mutant. Differences were evaluated by one-way ANOVA (mean ± SD from triplicate; *p < 0.05, **p < 0.01, ***p < 0.005 and ****p < 0.001). (**g**) R110/AFC ratio obtained from (**e**,**f**) Differences were evaluated by one-way ANOVA (mean ± SD from triplicate; ***p < 0.005 and ****p < 0.001). N.S. indicates not significant.

We next conducted time-lapse imaging to visualise the clearance of TDP-43 aggregates by 3B12A scFv-CMA in living cells. We used HEK293A cells for this purpose because the cytoplasmic areas of these cells are relatively wide, permitting clear visualisation of GFP-fused cytoplasmic TDP-43 aggregates. HEK293A cells overexpressing GFP-TDP-43^mNLS,C173S/C175S^ demonstrated a gradual increase in the number and size of cytoplasmic aggregates over 48-h post-transfection. Conversely, Hoechst counterstaining demonstrated a concomitant decline in live cells, indicating that these cytoplasmic TDP-43 aggregates are cytotoxic (Fig. 5a). In contrast, cultures co-transfected with 3B12A scFv-CMA and GFP-TDP-43^mNLS,C173S/C175S^ showed significantly fewer and smaller TDP-43 aggregates and more numerous Hoechst-stained (viable) cells at 48-h post-transfection, indicating reduced aggregation and thus rescued from cell death (Fig. 5a–d) (Supplementary Movie 1a,b).

We also conducted biochemical analysis of cytotoxicity and cell death in the neuroblastoma cell line N2a to evaluate whether 3B12A scFv can rescue neuronal cells as well as non-neuronal HEK292A cells. Neuroprotection against TDP-43 aggregates by 3B12A scFv was evaluated by simultaneous treatment of transfected N2a cells with membrane-impermeable bis-AAF-R110 and cell-permeable GF-AFC, fluorescent compounds that react with proteases in dead and live cells, respectively. Among the TDP-43 species tested, cytoplasmic TDP-43 aggregates (TDP-43^mNLS,C173S/C175S^) induced the highest cytotoxicity, consistent with our previous studies in the NSC-34 cell line^33^. This cytotoxicity was significantly reduced in the presence of 3B12A scFv (VH_VL), and the inclusion of the CMA signal significantly enhanced this protective effect (Fig. 5e–g), while 3B12A scFv species alone (vector controls) did not induce neurotoxicity or otherwise affect N2a cell viability.

### 3B12A scFv-CMA induces HSP70 in the presence of misfolded TDP-43 aggregates

The intrabody 3B12A scFv-CMA exhibited the high efficiency for clearance of cytoplasmic TDP-43 aggregates (Fig. 4d) and for reducing neurotoxicity induced by TDP-43 aggregates (Fig. 5f). The CMA is a selective proteolytic pathway associated with the heat shock protein 70 (HSP70) family member heat shock cognate protein 70 (HSC70)^39,40^, which functions as a molecular chaperone for the elimination of misfolded proteins. Therefore, we first examined if 3B12A scFv-CMA altered the expression of HSP70 family proteins including HSC70 and HSP70 in HEK293A cells.

**Figure 6:**
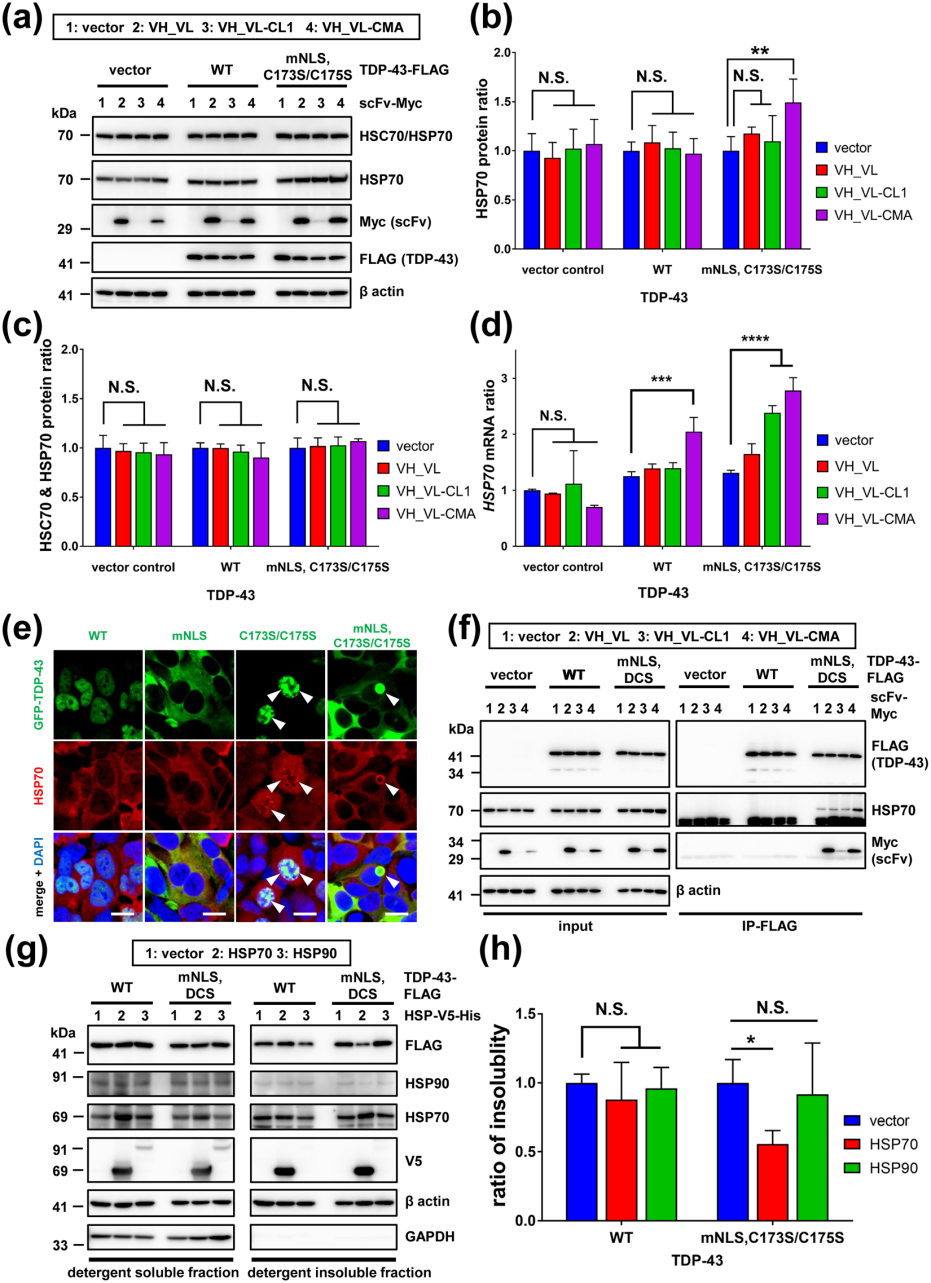
3B12A scFv-CMA induces HSP70 expression, which leads to the unfolding of TDP-43 aggregates. (**a–d**) Measurement of HSP70 expression in the presence of TDP-43 and 3B12A scFv. (**a**) Western blot analysis of endogenous HSP70 and HSC70 protein expression levels. (**b**,**c**) Quantified densitometric analysis of HSP70 protein ratio (**b**) and HSC70 and HSP70 protein ratio (designated as HSC70 and HSP70) obtained from immunoblots using an antibody recognising both HSC70 and HSP70 (N27F3-4) (**c**) Each data point was obtained by normalisation to actin. Differences were evaluated by one-way ANOVA (mean ± SD from three independent experiments; **p < 0.01 *vs* vector). (**d**) Quantitative real-time PCR analysis of *HSP70* gene expression. Differences were evaluated by one-way ANOVA (mean ± SD from triplicates; ***p < 0.005, ****p < 0.001 *vs* vector). N.S. indicates not significant. (**e**) Confocal microscopic analysis of HEK293A cells expressing GFP-fused TDP-43. Endogenous HSP70 colocalised with both nuclear and cytoplasmic TDP-43 aggregates (arrow heads), whereas HSP70 did not colocalise with TDP-43^WT^. Scale bar = 20 µm. (**f**) Interaction of endogenous HSP70 and overexpressed TDP-43 species. HSP70 interacted with cytoplasmic aggregated TDP-43 predominantly in the presence of VH_VL-CMA. DCS indicates the C173S/C175S mutant. (**g**) Western blot analysis for detergent insolubility of TDP-43 in the presence of HSP70 or HSP90 overexpression in HEK293A. Cell lysates were separated into RIPA-soluble or -insoluble fractions after centrifugation for 20 min at 15,000 × *g* at 4 °C and subsequently eluted in 2% SDS sampling buffer for 5 min at 95 °C. DCS represents the C173S/C175S mutant. (**h**) Quantified densitometric analysis of (**g**) Each data point was obtained by normalisation to actin. The insolubility ratio is normalised insoluble TDP-43 to normalised soluble TDP-43. Differences were evaluated by one-way ANOVA (mean ± SD from three independent experiments; *p < 0.05 *vs* vector).

Western blot analysis revealed that HSP70 was upregulated, but only when cells were cotransfected with 3B12A scFv-CMA and TDP-43^mNLS,C173S/C175S^ (Fig. 6a–c). In contrast, transfection had no effect on expression of HSC70. Quantitative real-time PCR analysis revealed that *HSP70* mRNA was significantly upregulated by TDP-43^mNLS,C173S/C175S^ and by 3B12A scFv-CMA in the presence of either cytoplasmic TDP-43 or the WT TDP-43. The scFv-CL1 also increased *HSP70* mRNA levels, but not HSP70 protein levels, only in the presence of TDP-43 aggregates. The induction of *HSP70* mRNA was highest in cells cotransfected with 3B12A scFv-CMA and TDP-43^mNLS,C173S/C175S^ (Fig. 6d). Immunofluorescence microscopy also revealed that endogenous HSP70 colocalised with nuclear and cytoplasmic TDP-43 aggregates in HEK293A cells (Fig. 6e). Consistent with immunofluorescence results, an immunoprecipitation assay showed that 3B12A scFv-CMA augmented the interaction of HSP70 with misfolded TDP-43 in HEK293A cells (Fig. 6f). Notably, Western blot analysis revealed that HSP70 overexpression significantly reduced the detergent insolubility of cytoplasmic aggregated TDP-43, suggesting initiation of refolding, while overexpression of another representative molecular chaperone, HSP90, had no effect on TDP-43 solubility (Fig. 6g,h). As expected, expression of the constitutive protein HSC70 remained stable as evidenced by immunoblot analysis using an antibody recognising both HSC70 and HSP70 (Fig. 6a,c). An HSC70-specific antibody was not available.

Taken together, these findings indicate that 3B12A scFv-CMA facilitates the proteolytic clearance of misfolded TDP-43 and that clearance is further augmented by the aggregate refolding effect of induced HSP70.

### 3B12A scFv-CMA reduces cytoplasmic TDP-43 inclusions in the murine foetal cerebral cortex following *in utero* electroporation

**Figure 7:**
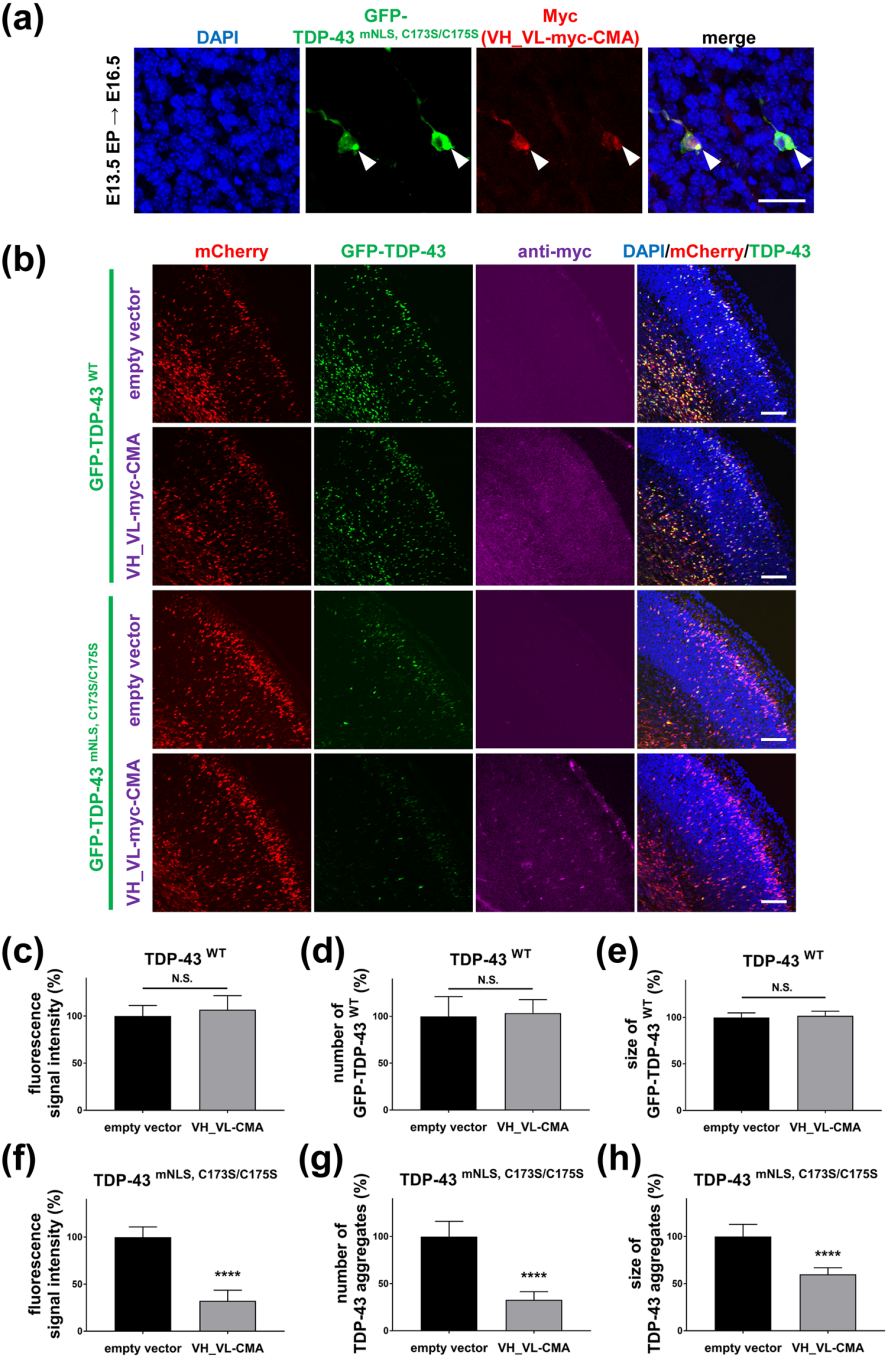
3B12A scFv-CMA reduces cytoplasmic TDP-43 aggregates in the murine cerebral cortex. (**a**) Myc-tagged 3B12A scFv-CMA colocalised with cytoplasmic TDP-43 aggregates in the cortical plate (CP, arrowheads). Scale bar = 50 μm. (**b**) Distribution of neurons expressing GFP-tagged WT and mutant TDP-43 in the E16.5 cortex. Electroporation was performed at E13.5, and brains were fixed at E16.5 followed by immunohistochemistry. Scale bar = 100 μm. (**c–h**) Quantification analysis of (**b**) TDP-43 aggregates were distinguished from non-aggregates by setting the fluorescence cut-off for the quantification using ImageJ software. Each data point was obtained by normalisation to mCherry. Differences were evaluated by unpaired *t*-test (mean ± SD, *n* = 7 for TDP-43^WT^ and n = 7 for TDP-43^mNLS,C173S/C175S^; ****p < 0.001 *vs* empty vector). N.S. indicates not significant.

*In utero* electroporation of murine embryonic brains is a well-established method to analyse the functions of exogenously overexpressed proteins *in vivo*^41–43^. To evaluate 3B12A scFv efficacy for the clearance of misfolded TDP-43 *in vivo*, we performed *in utero* electroporation to transduce TDP-43 alone or both TDP-43 and 3B12A scFv into murine brain. First, to investigate if the TDP-43^mNLS,C173S/C175S^ mutant also aggregates *in vivo*, we transduced GFP-TDP-43^mNLS,C173S/C175S^ plasmids into the cerebral cortex at E13.5, and the cortices were fixed at E16.5 for immunohistochemistry. We distinguished TDP-43 aggregates from nonaggregated (diffuse) TDP-43 by the distribution of GFP fluorescence^44,45^. Coronal brain sections exhibited GFP-TDP-43 mutant protein in both the lateral and medial neocortices, and the cellular distribution was grossly similar to that reported previously^41–43^ (Supplementary Fig. S7a). Histological examination of the lateral cortical plate (CP) (Supplementary Fig. S7b), together with enlarged images of the CP demonstrated that GFP-TDP-43^mNLS,C173S/C175S^ formed cytoplasmic aggregates in cells immunopositive for microtubule-associated protein 2a (MAP2a), a marker of mature neurons (Supplementary Fig. S7c,d). Some GFP-TDP-43^mNLS,C173S/C175S^ cytoplasmic aggregates in the CP were also immunoreactive to anti-phospho-TDP-43 (S409/S410) (Supplementary Fig. S7e) and to anti-ubiquitin antibodies (Supplementary Fig. S7f), consistent with the hyperphosphorylated and ubiquitinated status of aggregated TDP-43 in the spinal cord of ALS patients^5–9^. Consistent with our *in vitro* results in HEK293A cells, TDP-43^mNLS,C173S/C175S^ cytoplasmic aggregates colocalised with Myc-tagged 3B12A scFv-CMA *in vivo* (Fig. 7a).

To precisely evaluate the effects of 3B12A scFv-CMA on the clearance of misfolded TDP-43 *in vivo*, TDP-43 plasmids were electroporated into the cortex together with the 3B12A scFv-CMA or control plasmid plus mCherry expression vector to validate electroporation efficiency. As expected, GFP-TDP-43^WT^ distributed to nuclei in the CP and did not form detectable aggregates. Moreover, the relative fluorescence signal intensity, number and size of TDP-43^WT^ puncta in nuclei were not altered in the presence of 3B12A scFv-CMA compared with control cortices expressing GFP-TDP-43^WT^ alone (Fig. 7b–e). Alternatively, TDP-43^mNLS,C173S/C175S^ formed cytoplasmic puncta, which were markedly reduced in intensity, number and size by co-electroporation of 3B12A scFv-CMA (Fig. 7b,f–h).

To investigate whether 3B12A scFv-CMA expression affects early brain development, brains electroporated with the mCherry plasmid alone or plus the 3B12A scFv-CMA plasmid at E13.5 were subjected to immunohistochemistry at postnatal day 21 (P21). Similar to mice electroporated with the empty vector, those expressing 3B12A scFv-CMA were safely born at term, grew normally and exhibited age-appropriate behaviours until P21 (Supplementary Movie 2). Immunohistochemistry revealed that Myc-tagged 3B12A scFv-CMA distributed throughout the neocortex (Supplementary Fig. S8), while neuron counts were similar to those of vector controls as revealed by NeuN immunostaining (Supplementary Fig. S9a). There was no abnormal proliferation of GFAP-positive astrocytes or Iba1-positive microglia indicative of tissue damage and reactive inflammation (Supplementary Fig. S9b,c).

## Discussion

We demonstrate that 3B12A scFv-CMA can serve as an effective and selective proteolytic intrabody against intracellular misfolded TDP-43 in cultured cells and murine cerebral cortex. A scFv targeting the N-terminal amino acids adjacent to the expanded polyglutamine of huntingtin protein was previously shown to successfully prevent protein aggregation^46,47^, underscoring the broad potential of this intrabody strategy for control of neurodegenerative diseases due to proteinopathy. Similarly, a scFv against amyloid oligomers attenuated α-synuclein aggregation and improved motor and cognitive functions in a mouse model of Parkinson’s disease^48^. To the best of our knowledge, ours is the first study to evaluate the efficacy of a scFv intrabody with dual proteolytic signals for clearance of intracellular misfolded TDP-43 both *in vitro* and *in vivo*. Unexpectedly, the scavenging effect of CMA-fused intrabody was augmented by the aggregate refolding function of HSP70, which was transcriptionally induced upon aggregate−scFv binding (Fig. 6). The exact mechanism for *HSP70* mRNA induction by the interaction between scFv-CMA and aggregated substrates remains elusive. Considering that the expression of TDP-43 aggregates or CMA-scFv alone did not evoke measurable HSP70 induction, autophagy induction might also be required. HSP70 confers neuroprotection by refolding TDP-43^49^, and the HSP70 inducer arimoclomol was tested in a clinical trial of ALS patients up to phase II/III. Therefore, the unique double action of our 3B12A scFv-CMA, proteolysis and refolding, is an effective mechanism for molecular targeting and clearance of pathogenic proteins. Further, this strategy was highly specific to the pathogenic form, as 3B12A scFv-CMA interacted with TDP-43 ^mNLS^ whether aggregated or nonaggregated but significantly accelerated degradation only of the aggregated form.

The 3B12A scFv intrabody contains a PEST-like peptide on the VH domain, which may also be involved in proteasomal degradation^30–32^. The other function of the PEST-like peptide is to promote folding by altering the overall charge of the scFv to negative^50^. Indeed, the calculated net charges of 3B12A-VH-VL and 3B12A-VH-VL without the PEST-like sequence at pH 7 were only −3 and −1, respectively. The overexpression of CL1- or CMA-scFv with or without TDP-43 aggregates did not affect the autophagic or proteasomal activity. Although the sensitivities of proteasomal activity assays and autophagic flux assays are not high, results thus far suggest that binding to intrabodies with proteasome-targeting CL1 and lysosome-targeting CMA signals may be sufficient to clear TDP-43 aggregates without disrupting normal TDP-43 function or protein homeostasis. Previous studies have documented the efficacy of PEST-fusion intrabodies against pathogenic proteins such as huntingtin^46^ and α-synuclein^51^. It was also shown that intrabodies can disrupt the subcellular distribution of target proteins, including by inhibition of nuclear−cytoplasmic translocation^52,53^. Although cytoplasmic nonaggregated TDP-43 (TDP-43^mNLS^) was not degraded by 3B12A scFv in cultured cells (Fig. 4e), further evaluations of 3B12A scFv effects on pre-aggregated TDP-43 nuclear−cytoplasmic redistribution are required to assess the potential for pre-emptive therapy.

The 3B12A antibody targets D247 of TDP-43^27^, a residue masked in the physiological condition by its location inside the molecule. The next residue, E246, is recognised by the von Hippel Lindau (VHL)/cullin-2 ubiquitin ligase complex for misfolded TDP-43^29^. This explains why 3B12A scFv specifically interacted with mislocalised/misfolded TDP-43 but not with TDP-43^WT^ in cells (Fig. 2).

There is yet no model TDP-43 species replicating all pathological features of sporadic ALS. In this study, we used the C173S/C175S artificial mutant as a TDP-43 inclusion mimic based on evidence of structural similarity to aggregated TDP-43 inclusions in ALS^33^. Using high pressure NMR analysis, it was previously shown that C173 and C175 residues in the RRM1 domain are crucially involved in preserving the conformation of TDP-43, while disruption of the equilibrium between free and intramolecular disulfide states may cause irreversible TDP-43 aggregation. The structural similarity of this C173S/C175S mutant to TDP-43 inclusions was supported by immunohistochemistry using an antibody against the deformed loop (W113–T116) in RRM1^33^. The sustained mislocalisation of TDP-43 in the cytosol by the NLS mutation induces cell death and occasionally forms inclusions both *in vitro* and *in vivo*^34,54^. However, these aggregates are relatively small in number and rarely phosphorylated or ubiquitinated^34,54^; thus TDP-43^mNLS^ is an insufficient model for TDP-43 proteinopathy. Transgenic mice overexpressing human TDP-43^WT^ and several known FALS mutants such as A315T and Q331K under native promoters develop similar mild phenotypes for FTLD-motor neuron diseases, but nuclear exclusion and cytosolic aggregates are prominent only in the FALS mutant models^55^. We reported that both WT and NLS mutant TDP-43 induce cell toxicity in immortalised cultured cells^34^. It should be noted that in the current work and our previous study^33^, aggregate-prone TDP-43^mNLS,C173S/C175S^ induced greater cell toxicity than TDP-43^WT^ or TDP-43^mNLS^, irrespective of mislocalisation. The pathologies induced by this TDP-43 mutant share several crucial properties with TDP-43 proteinopathy in addition to the strong trend for aggregation, including the involvement of prion-like domains, impaired RNA splicing, ubiquitination and phosphorylation, as well as motor neuron toxicity^33^. Based on the extent of aggregate toxicity, the TDP-43^mNLS,C173S/C175S^ mutant may be the most robust form for modelling ALS-associated TDP-43 proteinopathy, especially for studies on therapeutic strategies and efficacy.

Most promising for clinical applicability, 3B12A scFv-CMA reduced cytoplasmic TDP-43 aggregates in the lateral CP of murine embryos (Fig. 7) without causing gross developmental defects. Indeed, mice expressing 3B12A scFv through *in utero* electroporation did not show aberrant/ectopic neuronal cells, gliosis or microglial activation compared with vector controls. Of course, the restricted distributions of exogenous human TDP-43 and 3B12A scFv-CMA protein in mice following *in utero* electroporation limit future experiments on mitigation of neurological behaviour phenotypes, and the environment of the pup brain is distinct from the aged human brain. However, the successful elimination of TDP-43 aggregates *in vivo* validates the basic principle and justifies further analyses in older disease model animals. In the clinical setting, our intrabody may be effectively delivered by a virus vector, such as an adeno-associated virus (AAV). Indeed, AAV9-mediated intrathecal delivery of a scFv derived from the D3H5 monoclonal antibody against misfolded SOD1 reduced misfolded SOD1 in the spinal cord, delayed disease onset and enhanced the survival of SOD1^G93A^ transgenic mice^56^. A recent study demonstrated that suppression of human TDP-43 mutant expression after disease onset in a mouse model decreased TDP-43 pathology, rescued motor deficits and prolonged survival^23^. These results provide strong support for our approach of pathological TDP-43 removal using a 3B12A intrabody, even after disease onset and formation of pathological TDP-43 aggregates. Further investigations evaluating the potential of our degradative 3B12A intrabody against pathogenic TDP-43 using appropriate animal models are required to advance this novel molecular targeting therapy for ALS.

## Methods

### Plasmid construction

In cell culture studies, mammalian expression plasmids for TDP-43 tagged with FLAG (pcDNA3-TDP-43-FLAG) or EGFP (pEGFP-N3-TDP-43) were constructed using a conventional PCR technique as described previously^34^. The TDP-43 substitution mutant with serine at Cys173 and Cys175 (C173S/C175S; DCS), familial ALS-linked mutations (A315T and Q331K) and mNLS (R82L/K83Q) were generated by site-specific mutagenesis^34^. A plasmid for Halo-tagged TDP-43 (WT and C173S/C175S; DCS) was generated by subcloning TDP-43 cDNA into pFC14A HaloTag® CMV Flexi® Vector (Promega, Fitchberg, WI). cDNA for variable fragments of the heavy chain (VH) and light chain (VL) were obtained using mRNA from a hybridoma producing 3B12A monoclonal antibody, which recognises mislocalised/misfolded TDP-43^27^. For *in utero* electroporation studies, target protein expression was driven by the CAGGS promoter^57^. Detailed protocols regarding construction and domain maps of Halo-tagged TDP-43 (TDP-43-Halo), CAGGS promoter-driven TDP-43, and single-chain variable fragment (scFv) for VH-VL-Myc, VL-VH-Myc, VH-Myc, VL-Myc, VH-VL-CL1-Myc and VH-VL-CMA-Myc are described in Supplementary Materials and Methods and Figure S1.

### Antibodies

Antibodies used in this work are described in the Supplementary Table.

### Cell culture and transfection

All cultured cells were maintained at 37 °C under 5% CO_2_ and 100% humidity. HEK293A (Invitrogen, Carlsbad, CA) and N2a cells (ATCC, Manassas, VA) were maintained in Dulbecco’s modified Eagle’s medium (DMEM; Nacalai, Kyoto, Japan) containing 10% foetal bovine serum (FBS) and penicillin/streptomycin (Nacalai). The FuGene HD Transfection Reagent (Promega) was used for plasmid transfection. At 48 h after transfection, cells were treated for various analyses as indicated.

### Western blotting and immunoprecipitation

Cultured cells were lysed in sodium dodecyl sulfate (SDS) buffer containing 2-mercaptoethanol for Western blotting or in immunoprecipitation assay (RIPA) buffer (20 mM HEPES-KOH [pH 7.4], 125 mM NaCl, 2 mM EDTA, 1% Nonidet-P40, 1% sodium-deoxycholate) containing protease inhibitor cocktail (Roche, Basel, Switzerland) for immunoprecipitation. Ten percent of each cell lysate volume was analysed as the total cell lysate, and the remaining 90% was incubated with anti-FLAG M2 affinity gel (Sigma, St. Louis, MI) or anti-Myc affinity beads (Nacalai) at 4 °C overnight. The affinity beads were subsequently washed with RIPA buffer five times, and the immunoprecipitates were eluted in 2% SDS sampling buffer for 5 min at 95 °C. The eluates were separated on polyacrylamide gels (Wako, Tokyo, Japan) and proteins transferred onto PVDF membranes (Millipore, Billerica, MA). Proteins were detected using an enhanced chemiluminescence system (ECL; Thermo-Fisher Scientific, Waltham, MA or Nacalai). Densitometric analysis of protein bands was performed using ImageJ software^58^.

### Immunofluorescence and microscopic analysis

Cultured cells were fixed in 4% paraformaldehyde (PFA)/PBS (pH 7.2) and permeabilised with 0.1% Triton-X100/PBS containing 5% normal goat serum as a blocking agent. Cells were reacted with primary antibody (4 °C, overnight) and subsequently with a fluorophore-tagged secondary antibody (Alexa; Invitrogen) for 1 h at room temperature. Cells were counterstained with 4′-6 diamidino-2-phenylindole (DAPI). Fluorescence images were obtained using a confocal laser microscope (FV1000-D IX81, Olympus, Tokyo, Japan).

### Sandwich ELISA

Target FLAG antibody in coating buffer (Roche) was coated onto ELISA plates (Nunc, Rochester, NY) at a 1:100 dilution. Coated FLAG antibody was then reacted with cell lysates from transfected HEK293A cells (prepared in RIPA buffer) for 1 h at room temperature and subsequently with anti-Myc antibody at a 1:500 dilution (4 °C, overnight). Then, the coated antibody was reacted with the peroxidase-conjugated secondary antibody (Jackson Immunoresearch, West Grove, PA). Finally, reaction buffer containing 2,2′-azino-bis-3-ethylbenzothiazoline-6-sulfonate (Roche) was applied, and the absorbance was measured by a spectrometer at 405 nm with a reference at 490 nm.

### HaloTag pulse chase assay

At 48 h after cotransfection of HEK293A cells, the HaloTag-fused protein was labelled with 1 μM diAcFAM ligand (Promega) in FBS-free culture medium for 15 min in a cell culture incubator. Cells were then washed three times with warm PBS and cell lysates prepared in SDS sample buffer at 0, 12 and 24 h after ligand treatment. All lysate samples were separated on polyacrylamide gels (Wako) by SDS-PAGE, and gels were subsequently analysed on a fluorescence scanner (LAS-3000; FUJIFILM, Japan).

### Time lapse imaging analysis

At 24 h after cotransfection, HEK293A cells were counterstained with Hoechst 33342 (Nacalai). Stained cells were observed at 30-min intervals over 48 h using a fluorescent microscope (BZX-710; Keyence, Osaka, Japan). For quantification of GFP-positive TDP-43 aggregates with ImageJ software, we removed background noise and set fluorescence thresholds of GFP fluorescence at 2.5% to distinguish TDP-43 aggregates from diffusely expressing cytoplasmic non-aggregated species and subsequently counted GFP-positive areas over 10 µm^2^ as aggregates. Intensity is expressed in arbitrary units.

### Protein degradation chase assay

Protein half-life in HEK293A cells was estimated starting 48 h after transfection by chronological chase analysis. Cells were treated with CHX (100 μg/ml) to inhibit protein synthesis. In several experiments, lactacystin (10 μM) or bafilomycin (0.1 μM) was added with CHX to inhibit proteasome or autophagy–lysosome activity, respectively. Dimethyl sulfoxide (DMSO) was used as a vehicle control.

### Measurement of cytotoxicity and cell viability

We estimated the cytotoxicity and viability of transfected N2a cells using the MultiTox-Fluor multiplex cytotoxicity assay kit (Promega). Briefly, N2a cells were seeded onto 96-well black culture plates and plasmids encoding FLAG-tagged WT or mutant TDP-43 and 3B12A scFv were co-transfected at 0.1 µg each per well. At 48 h after transfection, cell-permeant glycyl-phenylalanylamino fluorocoumarin (GF-AFC) and cell-impermeant bisalanyl-alanyl-phenylalanyl-rhodamine 110 (bis-AAF-R110), fluorescent indicators for live cells and dead cells, respectively, were applied. After a 2-h incubation, relative fluorescence emission was measured on a multi-plate reader (Perkin Elmer) with excitation/emission of 400/505 nm for live cells and 485/520 nm for dead cells.

### Quantitative real-time PCR

Total RNA samples were purified from HEK293A cells using a commercially available kit (Invitrogen) and converted to cDNA with reverse transcriptase (Invitrogen). The mRNA expression levels of *HSP70* and glyceraldehyde 3-phosphate dehydrogenase (*GAPDH*) were analysed using a real-time PCR Detection Systems (BIO-RAD, Hercules, CA) and SYBER quantitative PCR kit (Toyobo, Osaka, Japan) with the following primer pairs: *HSP70*, 5′-CAA GAT CAC CAT CAC CAA CG-3′ and 5′-TCG TCC TCC GCT TTG TAC TT-3′; *GAPDH*, 5′-GCA CCG TCA AGG CTG AGA AC-3′; and 5′-TGG TGG TGA AGA CGC CAG TGG A-3′. *GAPDH* was used as an internal standard, and the relative mRNA expression levels were calculated by the ΔΔCT method according to the manufacturer’s protocol using the included software (BIO-RAD).

### In utero electroporation

Timed-pregnant ICR mice (E13.5) were anaesthetised by inhalation of isoflurane (Pfizer, Tokyo, Japan) and intraperitoneal injection of sodium pentobarbital (Kyoritsu Seiyaku, Tokyo, Japan). The uterine horns were then exposed in the abdominal cavity. The expression vectors (1 μg/μl of GFP-tagged TDP-43 plasmids [pCAGGS-TDP-43^WT^-GFP or pCAGGS-TDP-43^mNLS,C173S/C175S^-GFP], 1 μg/μl of effector plasmids [pCAGGS-3B12A-VH_VL-Myc-CMA or empty plasmid], and 0.5 μg/μl of normalisation plasmid [pCAGGS-mCherry]) were mixed and diluted in PBS (−) containing 0.05% Fast Green, and 1–2 μl of the solution was injected into the lateral ventricles of embryos. After plasmid injection, embryo heads were held using a tweezer-type electrode (CUY650P5; Nepagene, Chiba, Japan) with the anode on the forebrain side in which the plasmid was injected, and five cycles of electric pulses (31 V, 50 ms, at 950-ms intervals) were delivered by an electroporator (NEPA21; Nepagene). The uterus was repositioned in the abdominal cavity, the skin sutured and pregnancy allowed to continue.

### Brain slice preparation and immunohistochemistry

Embryos were anaesthetised on ice and postnatal mice by intraperitoneal injection of sodium pentobarbital and isoflurane inhalation. Brains were fixed by transcardial perfusion with 4% paraformaldehyde (PFA) in 0.1 M PBS. The brains were immersed in 4% PFA at 4 °C overnight, incubated in 20% sucrose/0.1 M PBS at 4 °C overnight, embedded in OCT compound (Sakura Finetek, Japan), and frozen in liquid nitrogen. The restricted distribution of the plasmids in the dorsolateral cortex of the pup mice by *in utero* transporation allowed us only limited slices in serial sectioning. We therefore carefully tried to obtain adjacent areas. Frozen sections were cut coronally and mounted on glass slides (Matsunami, Osaka, Japan). The slides were washed with 0.1 M PBS/0.1% Triton X-100 (PBS-T) and blocked with 3% bovine serum albumin (Nacalai) in PBS-T at room temperature for 30 min. After subsequent incubation with primary antibody at 4 °C overnight, slides were incubated in Alexa Fluor-conjugated antibody (Invitrogen) at room temperature for 1 h, washed with PBS-T, and placed onto cover glass (Matsunami) with mounting medium containing DAPI for nuclear counterstaining (Vector Laboratories, Burlingame, CA). For quantification of GFP-positive TDP-43 aggregates in the lateral cortical plates with ImageJ software, we removed background noise and set fluorescence thresholds of GFP fluorescence at 1% to distinguish TDP-43 aggregates from diffusely expressing cytoplasmic non-aggregates, and subsequently counted GFP-positive areas over 20 µm^2^ as aggregates. Intensity is expressed in arbitrary units.

### Statistical analysis

Multiple group means were compared by one-way ANOVA with post hoc Tukey’s multiple comparison tests for pair-wise comparisons. Factor estimation in the two chronological data groups was evaluated by two-way ANOVA using Prism software (GraphPad, La Jolla, CA). A *p* < 0.05 was considered statistically significant.

### Ethics

The protocols for both genetic transformation experiments and animal experiments were approved by and performed under the guidelines of the Kyoto University Graduate School of Medicine ethics committee (#130181 for the genetic transformation experiments) and the Shiga University of Medical Science (#28-7 and #28–38 for the genetic transformation experiments, #2016-11-12 H for the animal experiments).

## Electronic supplementary material

Supplementary Movie 1a

Supplementary Movie 1b

Supplementary Movie 2

Supplementary information
