# Supplementary material for: Elimination of TDP-43 inclusions linked to amyotrophic lateral sclerosis by a misfolding-specific intrabody with dual proteolytic signals
Source: Sci Rep. 2018 Apr 16;8:6030. doi: 10.1038/s41598-018-24463-3 (PMC5902603; doi:10.1038/s41598-018-24463-3)
Supplement: Supplementary file 4 — Supplementary information [file 41598_2018_24463_MOESM4_ESM.pdf]

SUPPLEMENTARY MATERIALS

Elimination of TDP-43 inclusions linked to amyotrophic lateral sclerosis by a misfolding-specific intrabody with dual proteolytic signals

Yoshitaka Tamaki[1,2], Akemi Shodai[1], Toshifumi Morimura[3], Ryota Hikiami[1,2], Sumio Minamiyama[1,2], Takashi Ayaki[2], Ikuo Tooyama[3], Yoshiaki Furukawa[4], Ryosuke Takahashi[2], Makoto Urushitani[1,3*]

1. Department of Neurology, Shiga University of Medical Science, Otsu, Japan
2. Department of Neurology, Kyoto University Graduate School of Medicine, Kyoto, Japan
3. Molecular Neuroscience Research Center, Shiga University of Medical Science, Otsu, Japan
4. Department of Chemistry, Keio University, Yokohama, Japan

Plasmid construction

Halo-tagged TDP-43 was generated by PCR using WT or C173S/C175S; DCS TDP-43 as a template, using the primer pairs 5'- TAT AGG GCT AGC GAT ATG TCT GAA TAT ATT CGG GT-3' and 5'-TCA GTG GTT GGC TCG AGC ATT CCC CAG CCA GAA GAC -3', and subcloned into pFC14A HaloTag® CMV Flexi® Vector (Promega) at NheI/XhoI sites. For in utero electroporation studies, cDNA of VH-VL-CMA-Myc or TDP-43-EGFP (WT or mNLS, C173S/C175S) was amplified by PCR, and was subcloned into pCAGGS vector (RIKEN, BRC Bank) at NheI/Not I sites. The primer pairs are 5'-GGG GCT AGC CAC CAT GGA GGT TCA GCT GCA GCA GTC-3' and 5'- CCC GCG GCC GCT TAT TGT TCT CTG AAT TTC AGG TCC-3'for VH-VL-CMA-Myc, and 5'- GGG GCT AGC GCC ACC ATG TCT GAA TAT ATT CGG GT-3' and 5'- CCC GGA TCC CGC ATT CCC CAG CCA GAA GAC T-3', respectively. For generation of plasmids encoding 3B12A scFv, mRNA was purified from 3B12A hybridoma cells using a commercially available mRNA isolation kit (Invitrogen, Carlsbad, CA, USA), and cDNA was synthesised using SuperScript III Reverse Transcriptase (Invitrogen) with oligo-dT priming according to the manufacturer's protocol. The first-strand cDNAs encoding variable fragment regions of the heavy chain (VH) and light chain (VL) were amplified separately using the forward primer 5'-GAC TCG AGT CGA CAT CGA TTT TTT TTT TTT TTT TT-3' and the reverse primer 5'-CTC AAT TTT CTT GTC CAC CTT GGT GC-3' for VH, and the forward primer 5'-GAC TCG AGT CGA CAT CGA TTT TTT TTT TTT TTT TT-3' and the reverse primer 5'-CTC ATT CCT GTT GAA GCT CTT GAC AAT GGG-3' for VL. The PCR products encoding VH and VL were reacted to TOPO TA cloning vector (Invitrogen) and agarose gel-purified. The VH and VL domains were assembled and linked together with a flexible 15-amino acid linker (GGGGS ×3) as follows. The double strand nucleotides were synthesised coding GGGS x 3 linker (5'-GGT GGA GGC GGT TCA GGC GGA GGT GGC TCT GGC GGT GGC GGA TCT-3' and 5'-AGA TCC GCC ACC GCC AGA GCC ACC TCC GCC TGA ACC GCC TCC ACC-3'). Then, reverse primer spanning VH and the linker (5'-GGC GGT GGC GGA TCT GAG GTT CAG CTG CAG CAG T-3'), and the forward primer spanning the linker and VH, were annealed, which was used as a template for PCR generating VH-linker-VL cDNA, which was subcloned into pCMV-Myc vector (Clonetech) at EcoRI/KpnI sites, and subsequently subcloned into pcDNA3 at EcoRI/XhoI. The primer pair used here are 5'-GCC CAG GCC CGA ATT CGC CAT GGA GGT TCA GCT GCA GCA GT-3' and 5'-AGC TTC TGC TCG CCG GTA CCT ATT TCC AAC TTT GTC CCC-3'. To generate VL-VH-Myc plasmids, the following primers were used with the same protocols described above; 5'-GGC GGT GGC GGA TCT GAA ATT GTG CTC ACC CAG T-3' and 5'-TGA ACC GCC TCC ACC GGA GAC TGT GAG AGT GGT G-3' for annealing primers to link VL-linker and linker-VH, respectively. 5'-GCC CAG GCC CGA ATT CGC CAT GGA AAT TGT GCT CAC CCA GT-3' and 5'-AGC TTC TGC TCG CCG GTA CCT ATT TCC AAC TTT GTC CCC-3' were used for VL-linker-VH, to be subcloned into pCMV-Myc vector at EcoRI/KpnI. To generate VH-Myc and VL-Myc, cDNA for VH or VL cDNA were used for PCR as templates, using the following primer pairs; 5'-GCC CAG GCC CGA ATT CGC CAT GGA GGT TCA GCT GCA GCA GT-3' and 5'-AGC TTC TGC TCG CCG GTA CCG GAG ACT GTG AGA GTG GTG-3' for VH-Myc, 5'-GCC CAG GCC CGA ATT CGC CAT GGA AAT TGT GCT

CAC CCA GT-3' and 5'-AGC TTC TGC TCG CCG GTA CCT ATT TCC AAC TTT GTC CCC-3' for VL-Myc to be subcloned into pCMV-Myc at EcoRI/KpnI sites, and subsequently transferred to pcDNA3 at EcoRI/XhoI sites. The peptide sequence ACKNWFSSLSHFVIHL for protein disassembling signal CL1 or KFREQ for CMA was subcloned after Myc-tag by in-fusion enzyme reaction. KFREQ peptide sequence for CMA was constructed in this order based on the rule that Q could be located at the beginning or at the end of the sequence and that there could be up to two of the allowed hydrophobic residues (I, F, L or V) or two of the allowed positive residues (R or K), but only one negative charge provided either by E or D [59]. The reverse primers used for proteolytic signals are 5'- TAG ATG CAT GCT CGA GTT ACA GGT GGA TCA CGA AGT GGC TCA GGC TGC TGA ACC AGT TCT TGC AGG CCA GGT CCT CCT CTG AGA TC-3'for CL1, and 5'-TAG ATG CAT GCT CGA GTT ATT GTT CTC TGA ATT TCA GGT CCT CCT CTG AGA TC-3' for CMA. The forward primer for both signals was 5'-CAG TGT GCT GGA ATT CGC CAT GGA GGT TCA GCT GC-3'. PCR products were ligated into pcDNA3 at EcoRI/XhoI sites.

Measurement of proteasome activity.

Chymotrypsin-like activity was measured using a commercially available kit (Cayman Chemical Company, Ann Arbor, MI, USA). HEK293A cells were seeded in 96-well culture dishes. Lysates were analysed for proteasome activity by measuring 480 nm emission from 360 nm excitation on a multi-plate reader (Perkin Elmer, Waltham, MA, USA).

LDH and MTT assay.

We estimated cytotoxic effects of CHX, lacacystin and bafilomycin on HEK293A cells using LDH and MTT assay. HEK293A cells were seeded onto 96-well culture plates at the concentration of 5 x 10^5 cells/ml. At 48 h after incubation at 37 °C under 5% CO_2 and 100% humidity, cells were treated with each drugs at different concentrations for 10 hours. Sodium azide (NaN_3) was used for positive control. LDH and MTT assay were subsequently analysed using Cytotoxicity LDH Assay Kit-WST (Dojindo Molecular Technologies, Kumamoto, Japan) and MTT assay kit (Nacalai), respectively, according to the manufacture's protocols.

Reference

59. Cuervo AM. Chaperone-mediated autophagy: selectivity pays off. *Trends Endocrinol. Metab.* **21**, 142-150 (2010).

Supplementary Table

Antigen (clone)	Company	Origin	ICC	IHC	IB
FLAG (M2)	SIGMA	mouse	1:500	N.A.	1:1000
Myc (71D10)	Cell Signaling	rabbit	1:200	1:250	1:1000
TDP-43	Proteintech	rabbit	N.A.	N.A.	1:1000
β actin (C4)	Santa Cruz	mouse	N.A.	N.A.	1:1000
LC3	MBL	rabbit	N.A.	N.A.	1:1000
HSC70/HSP70 (N27F3-4)	Enzo	mouse	N.A.	N.A.	1:1000
HSP70 (6B3)	Cell Signaling	rat	1:50	N.A.	1:1000
HSP90	Proteintech	rabbit	N.A.	N.A.	1:1000
V5	Hytest	mouse	N.A.	N.A.	1:5000
GAPDH	Santa Cruz	rabbit	N.A.	N.A.	1:1000
MAP2a (AP20)	Invitrogen	mouse	N.A.	1:200	N.A.
Phospho-TDP-43 (S409/S410)	Cosmobio	rabbit	N.A.	1:250	N.A.
Ubiquitin (K48 linked)	Millipore	rabbit	N.A.	1:250	N.A.
NeuN (A60)	Millipore	mouse	N.A.	1:250	N.A.
GFAP	SIGMA	mouse	N.A.	1:500	N.A.
Iba1	Wako	rabbit	N.A.	1:500	N.A.

Antibody information used in this work.
ICC, immunocytochemistry; IHC, immunohistochemistry; IB, immunoblotting.
N.A. indicates "not assessed".

Supplementary Figure S1

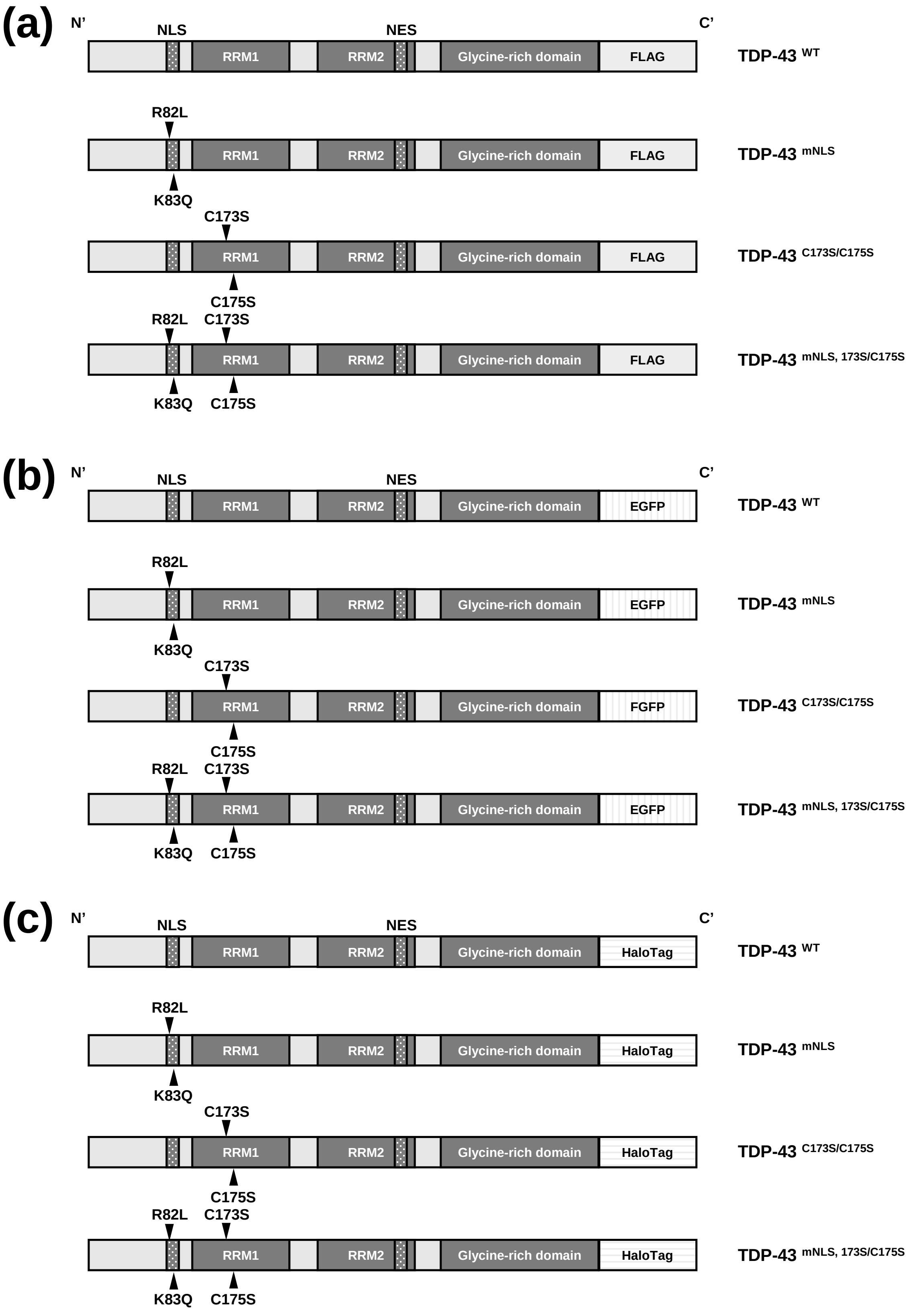

Illustrative domain profiles of TDP-43 constructs.
a–c. Domain structures of TDP-43 constructs with FLAG tag **(a)**, EGFP **(b)**, or HaloTag **(c)** at C-terminus were shown. Arrow heads indicate the amino acid profiles of experimental mutations.

Supplementary Figure S2

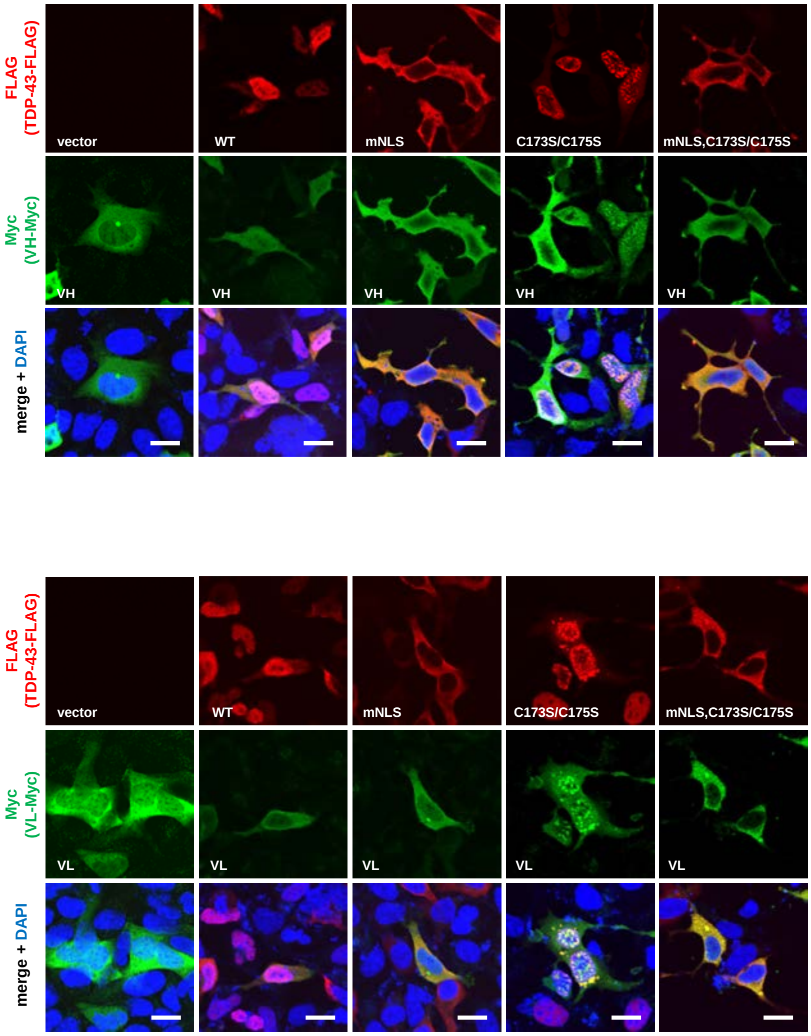

3B12A nanobodies colocalise with misfolded and mislocalised TDP-43.
a, b. Confocal laser micrographs of HEK293A cells expressing TDP-43-FLAG (red) and a Myc-tagged 3B12A nanobodies (green) at 48 h after transfection. DAPI was used for counterstaining of nuclei (blue). 3B12A nanobodies (VH or VL) colocalised with TDP-43 ^{mNLS}, TDP-43 $^{C173S/C175S}$ and TDP-43 $^{mNLS, C173S/C175S}$. Scale bar = 20 µm.

Supplementary Figure S3

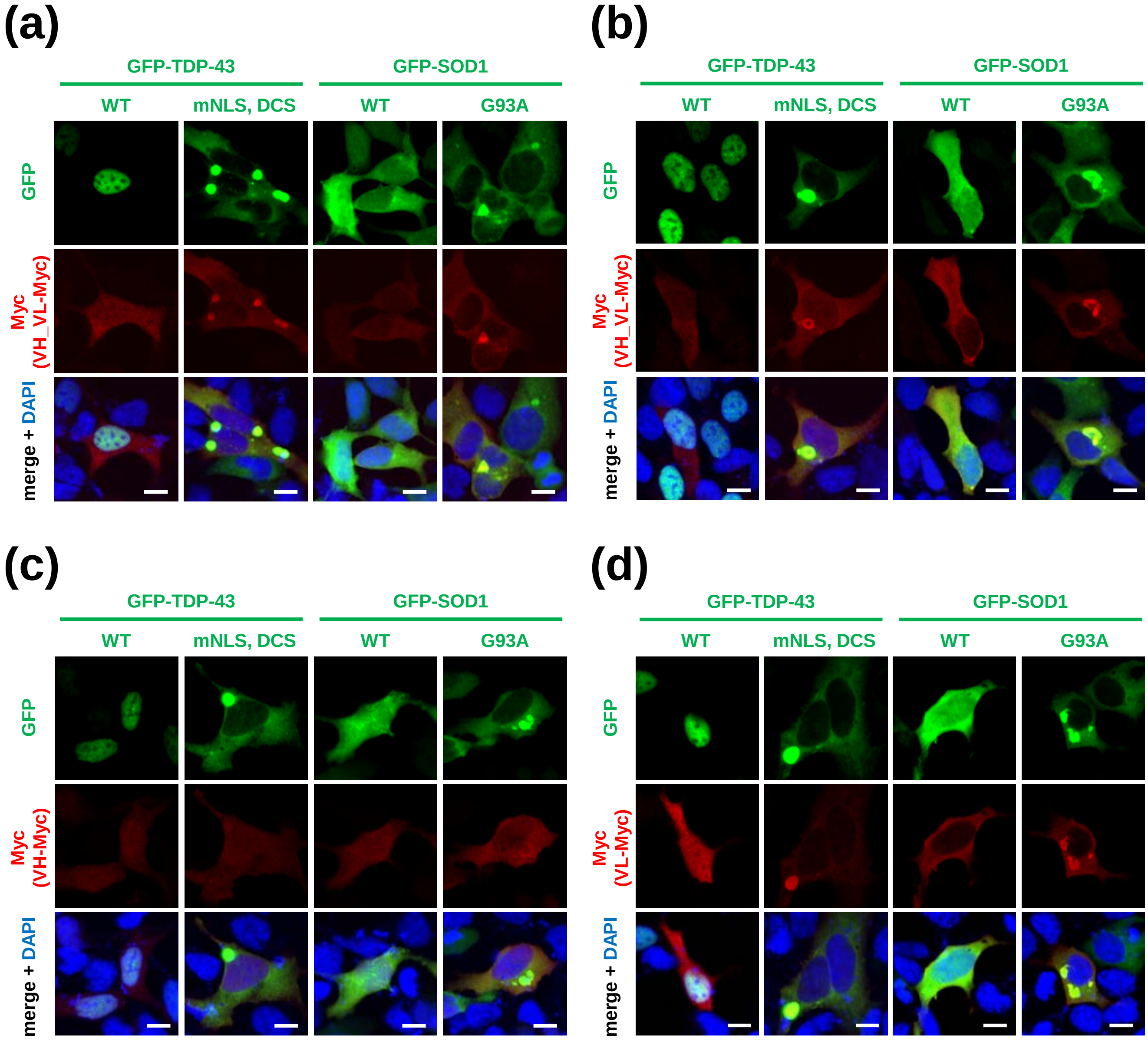

3B12A intrabodies partially colocalise with SOD1 ^{G93A} inclusions.
a–d. Confocal microscopic analysis of HEK293A cells overexpressing GFP-fused TDP-43 or SOD1 species and Myc-tagged 3B12A intrabodies. DCS represents the C173S/C175S mutant. Scale bar = 20 µm.

Supplementary Figure S4

(a) (b)

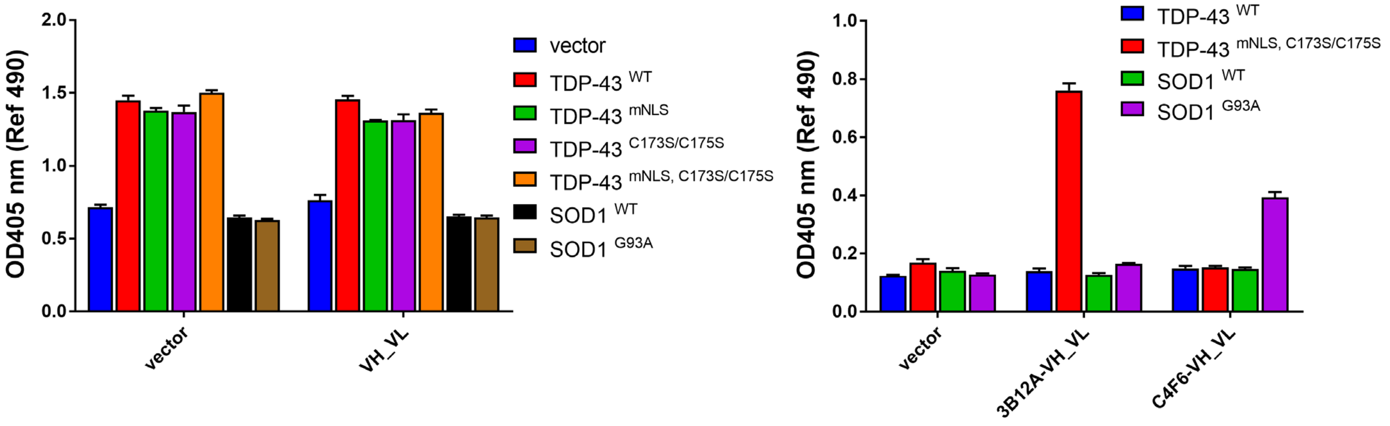

3B12A scFv has high affinity against misfolded TDP-43 compared to C4F6 scFv against SOD1 ^{G93A} mutant.

a, b. Co-transfected HEK293A cell lysates with Myc-tagged 3B12A scFv (VH_VL) and FLAG-tagged TDP-43 or SOD1 species were reacted to ELISA plates coated with anti-FLAG antibody, and subsequently detected with anti-TDP-43 antibody **(a)**, or with anti-Myc antibody **(b)**.

Supplementary Figure S5

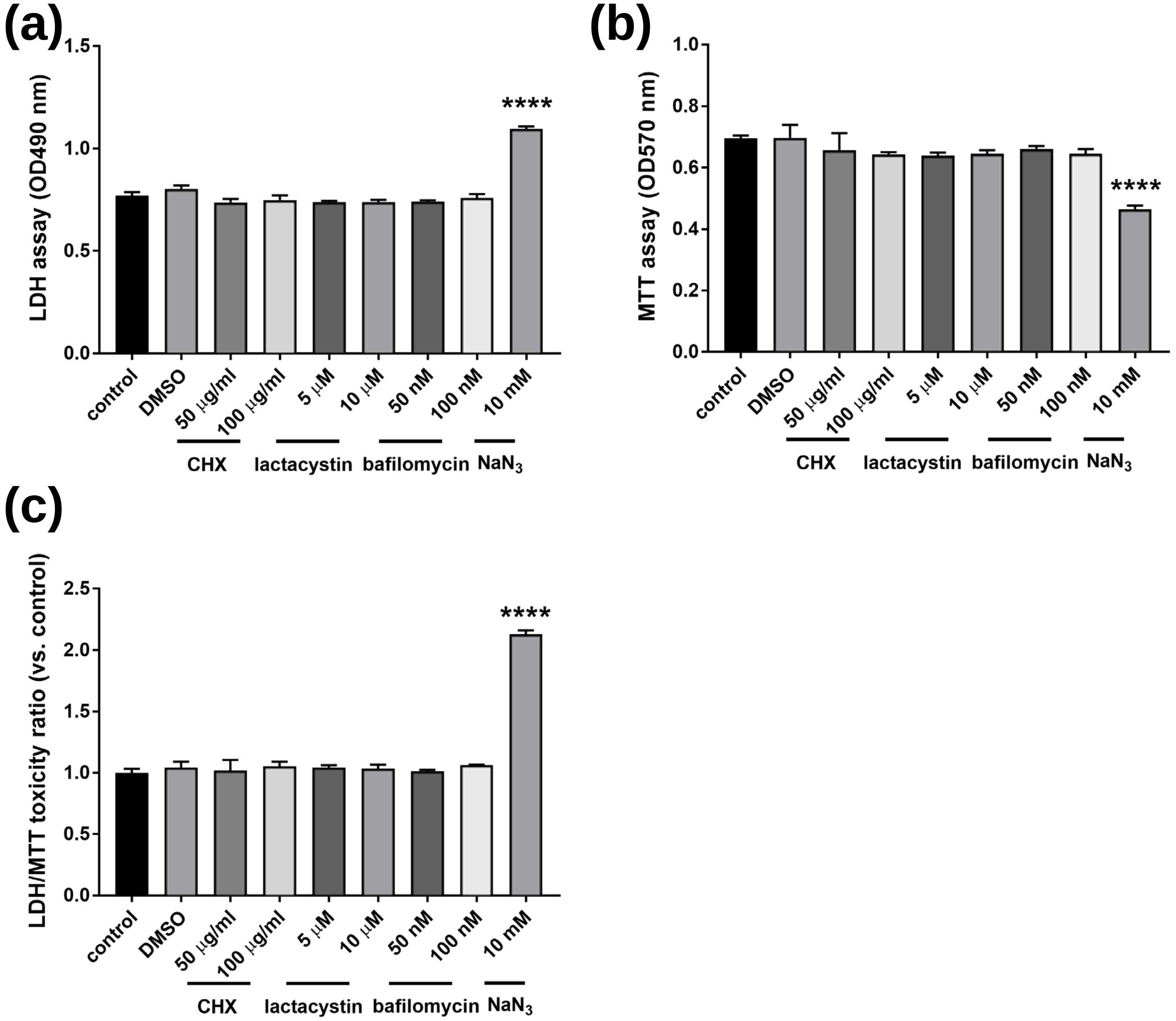

Cycloheximide (CHX), lactacystin and bafilomycin do not affect on cytotoxicity.
a, b. HEK293A cells were treated to CHX, lactacystin or bafilomycin for 10 hours before LDH **(a)** or MTT assay **(b)**. Untreated cells were used for control, and sodium azide (NaN_3) was for positive control.
c. LDH/MTT ratio obtained from **(a)** and **(b)** was calculated and evaluated for the toxicity. Differences were evaluated by one-way ANOVA (mean ± SD from triplicates; ****p < 0.001 *versus* control).

Supplementary Figure S6

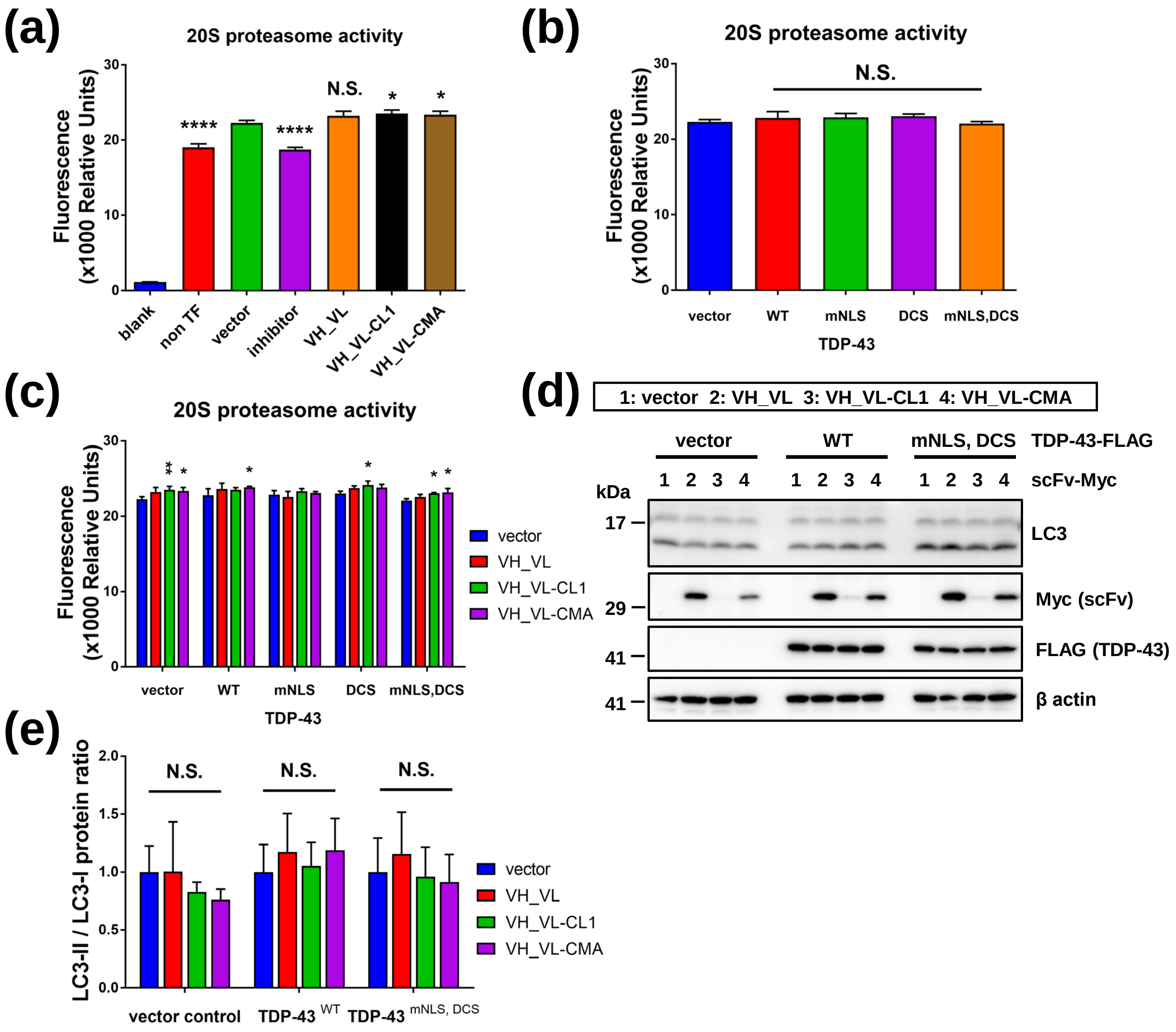

3B12A scFv does not repress either proteasome or autophagic activity.

a, b. Effect of overexpression of either 3B12A scFv or TDP-43 species on proteasome activity. Differences were evaluated by one-way ANOVA (mean ± SD from triplicates; **p* < 0.05, *****p* < 0.001 *versus* vector control). TF indicates transfection. DCS represents the C173S/C175S mutation. Inhibitor indicates Epigallocatechin Gallate (EGCG), a control inhibitor for proteasome.

c. Effects of both 3B12A scFv and TDP-43 species overexpression on proteasome activity. Differences were evaluated by one-way ANOVA (mean ± SD from triplicates; **p* < 0.05, ***p* < 0.01 *versus* vector control).

d. Effects of TDP-43 species and 3B12A scFv on the autophagy flux. Autophagy–lysosomal protein degradation activity was evaluated by measuring LC3 ratio (LC3-II/LC3-I). LC3-I and LC3-II represent upper and lower LC3 immunoblotted band, respectively.

e. Quantified analysis of LC3-II/LC3-I protein ratio in **(d)**. Each data point was obtained by normalization to actin. Differences were evaluated by one-way ANOVA (mean ± SD from three independent experiments; *versus* vector). N.S. indicates not significant.

Supplementary Figure S7

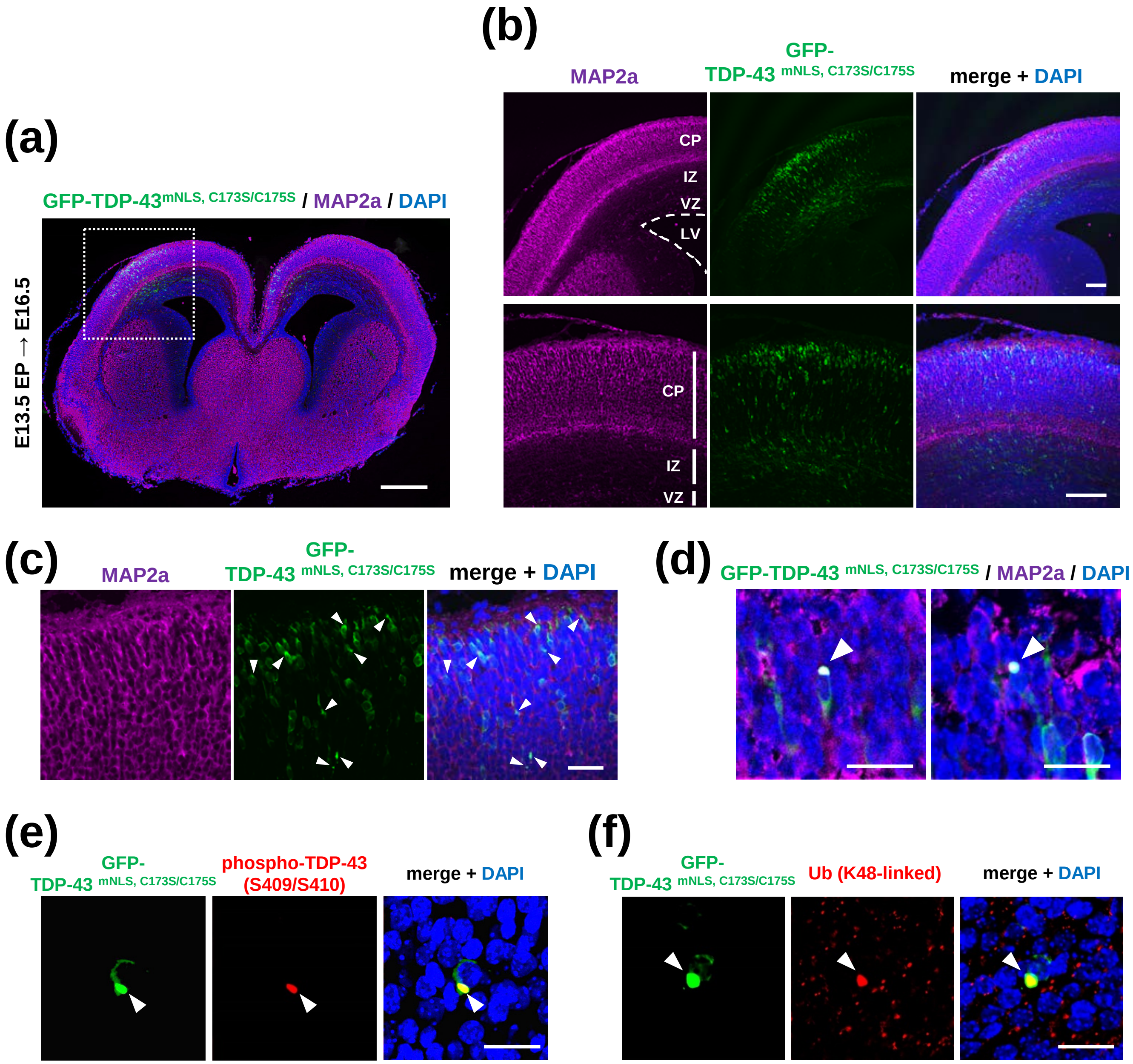

The TDP-43 $^{\text{mNLS, C173S/C175S}}$ mutant forms phosphorylated and ubiquitinated cytoplasmic aggregates in murine embryonic cerebral cortex.

a, b. Pyramidal neurons at E16.5 following electroporation with the GFP-TDP-43 $^{\text{mNLS, C173S/C175S}}$ gene at E13.5, pass by the intermediate zone (IZ) and reach the top of the cortical plate (CP). VZ and LV indicate the ventricular zone and lateral ventricle, respectively. Scale bar = 500 μm **(a)**, and = 200 μm **(b)**.

c, d. GFP-TDP-43 $^{\text{mNLS, C173S/C175S}}$ formed cytoplasmic aggregates in MAP2a-positive mature neurons in the CP (arrow heads). Scale bar = 50 μm.

e, f. GFP-TDP-43 $^{\text{mNLS, C173S/C175S}}$ cytoplasmic aggregates in the CP were immunostained with anti-phospho-TDP-43 (S409/S410) **(e)** and anti-ubiquitin (K48-linked) **(f)** antibodies (arrow heads). Scale bar = 50 μm.

Supplementary Figure S8

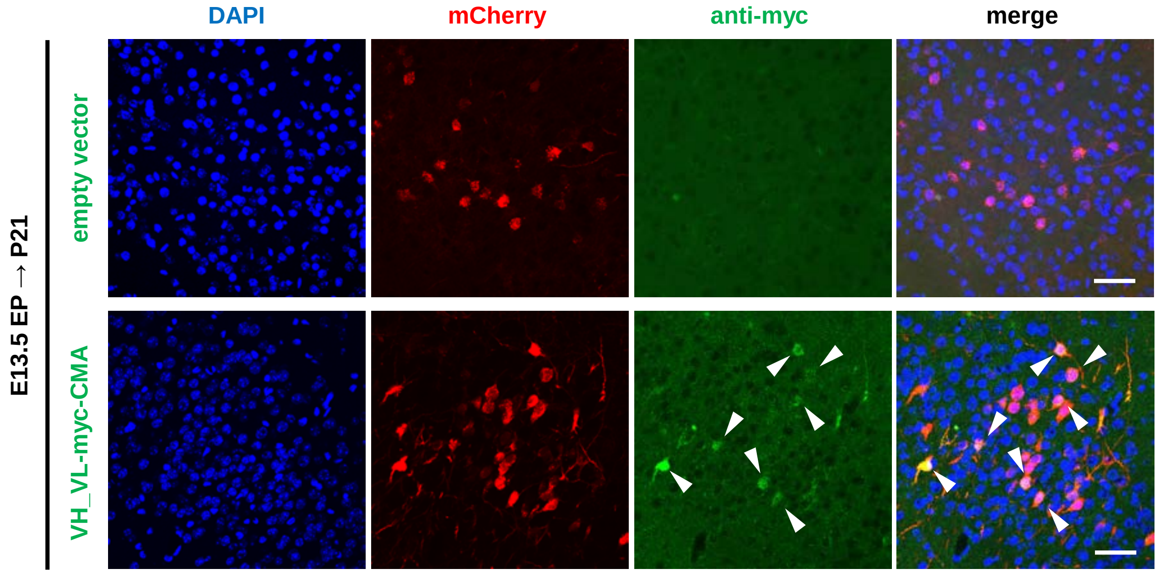

3B12A scFv-CMA distributes in the cortex of *in utero* electroporated P21 mice. Electroporation was performed at E13.5 and brains were fixed at P21 followed by immunohistochemistry. Myc-tagged 3B12A scFv-CMA was detected with mCherry in the cerebral neocortex of *in utero* electroporated P21 mice. Scale bar = 50 μm.

Supplementary Figure S9

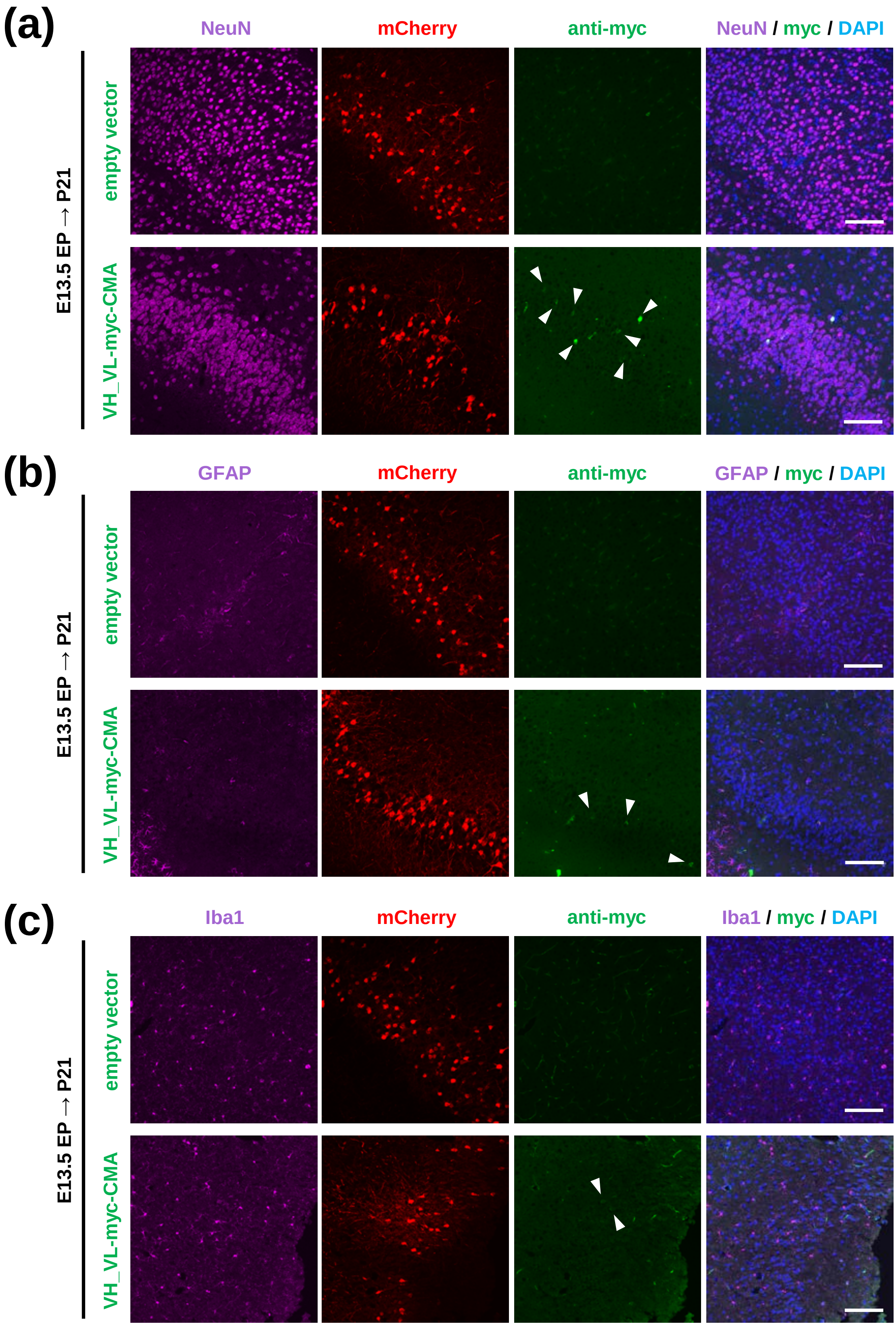

3B12A scFv-CMA does not affect early neural development in *in utero* electroporated mice.

a–c. Electroporation was performed at E13.5 and brains were fixed at P21 followed by immunohistochemistry. The P21 electroporated murine cortex was intensely stained by NeuN **(a)**, but not GFAP **(b)** or Iba1 **(c)** antibodies. Arrow heads indicate Myc-tagged 3B12A-scFv-CMA. Scale bar = 100 µm.

Supplementary Figure S10

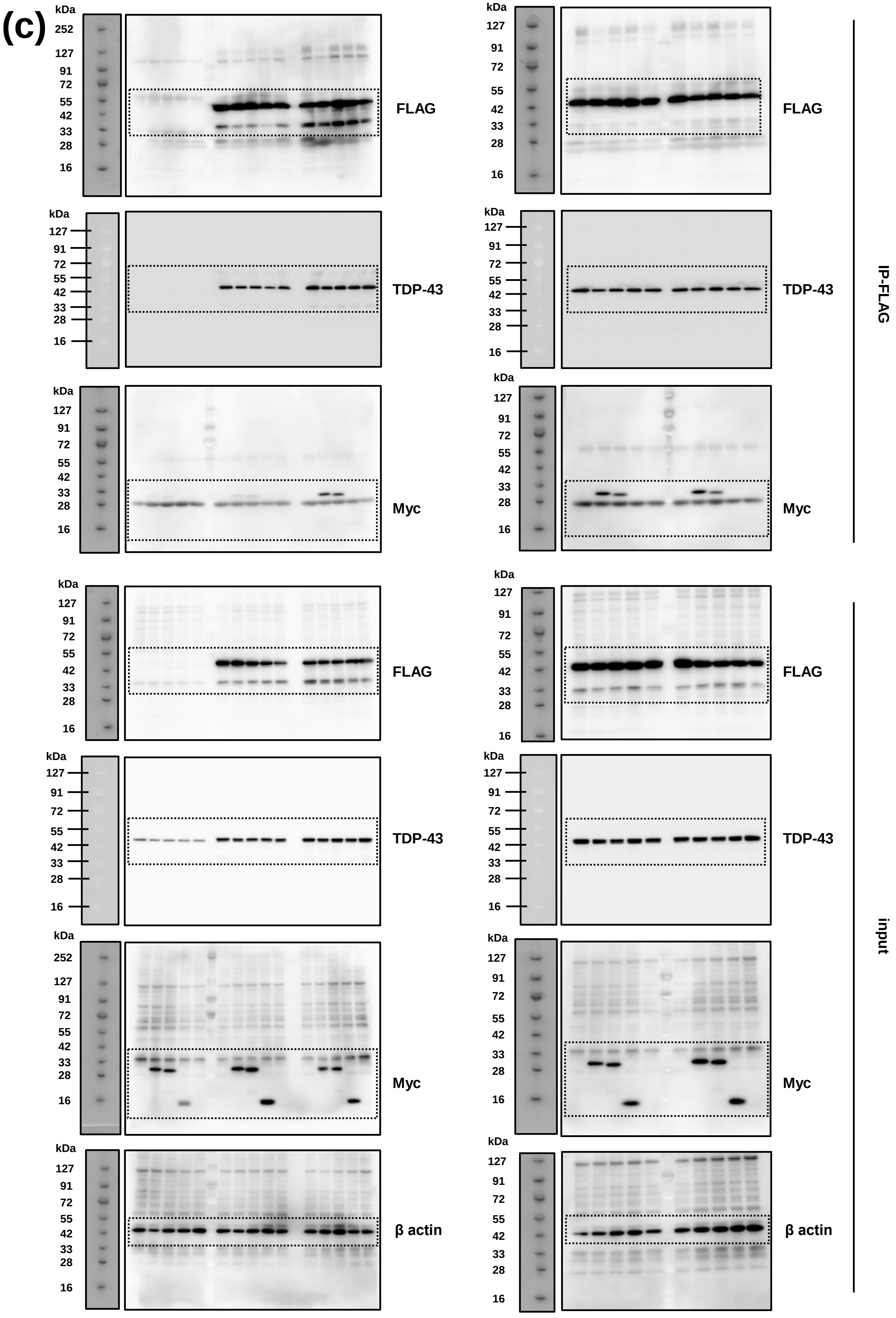

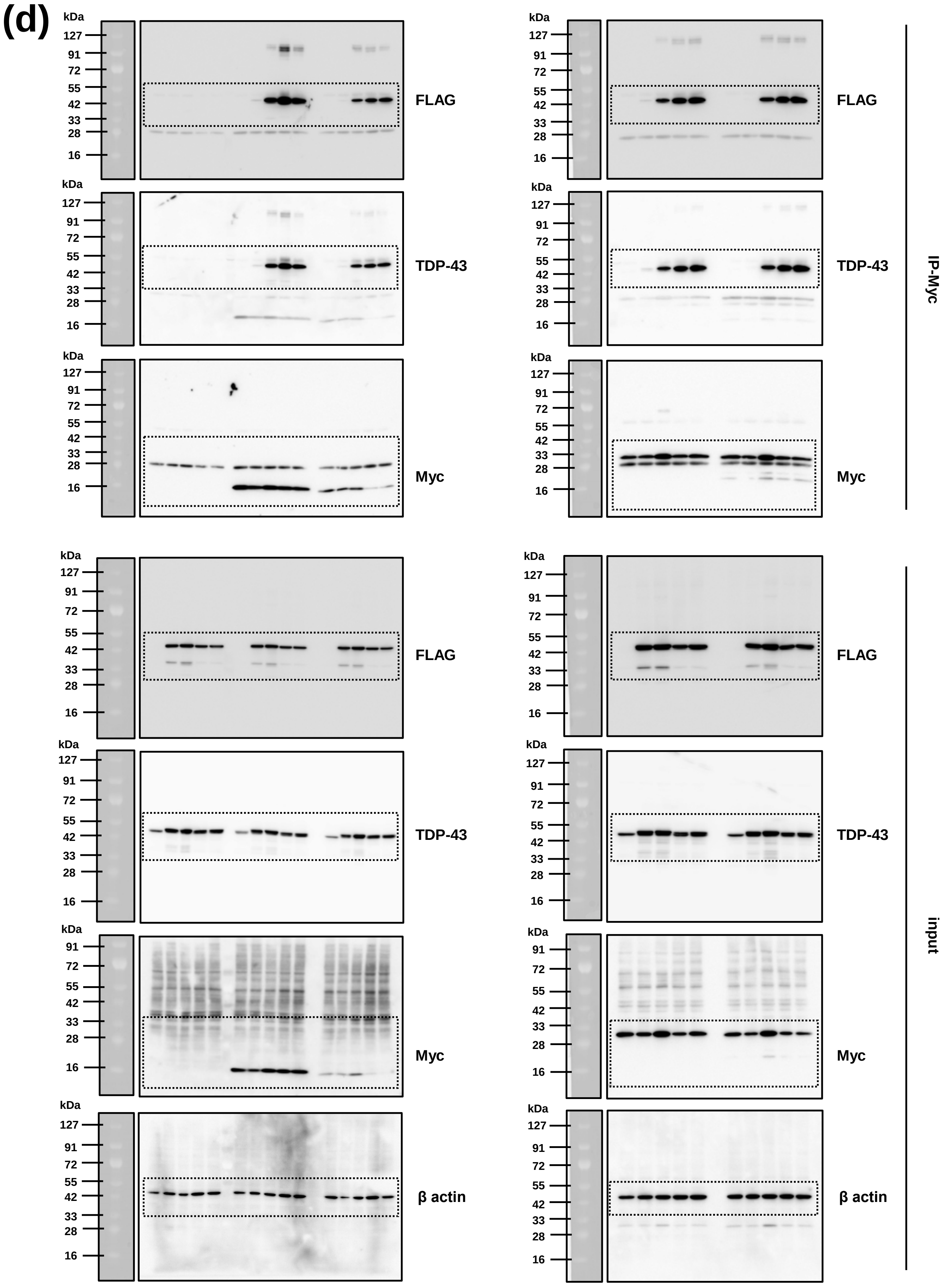

Original Western blots of trimmed panels in Figure 2.

Supplementary Figure S11

(b)

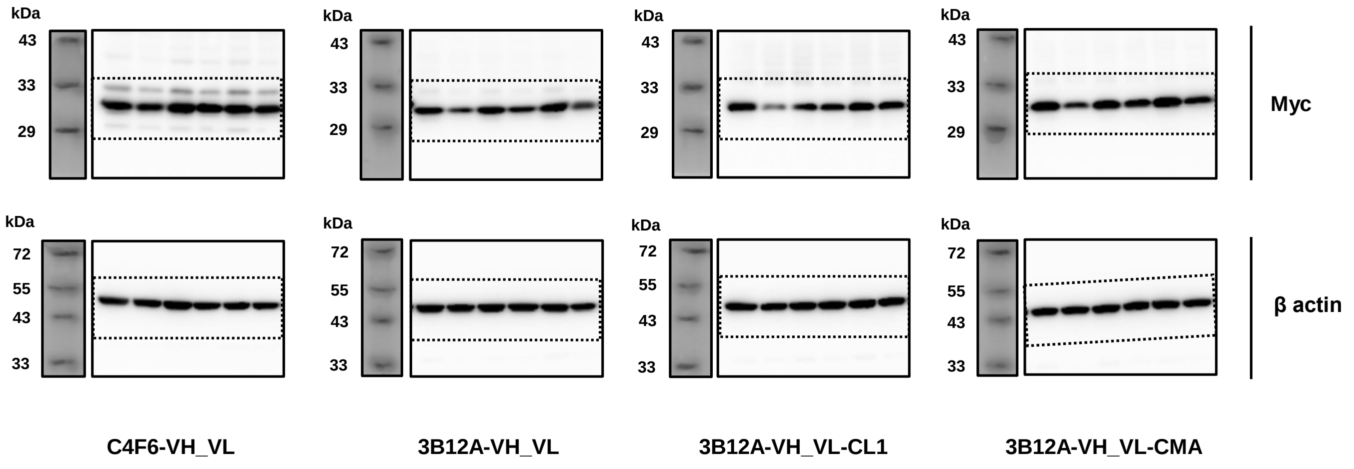

(d)

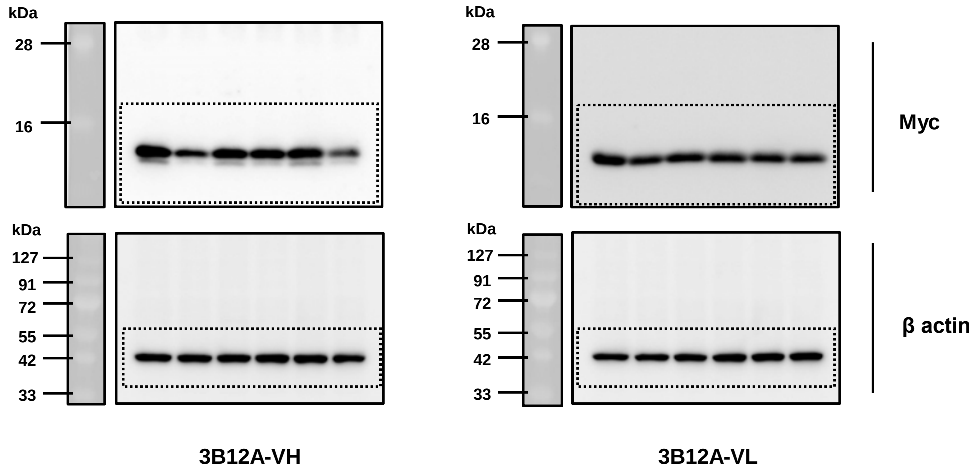

Original Western blots of trimmed panels in Figure 3.

Supplementary Figure S12

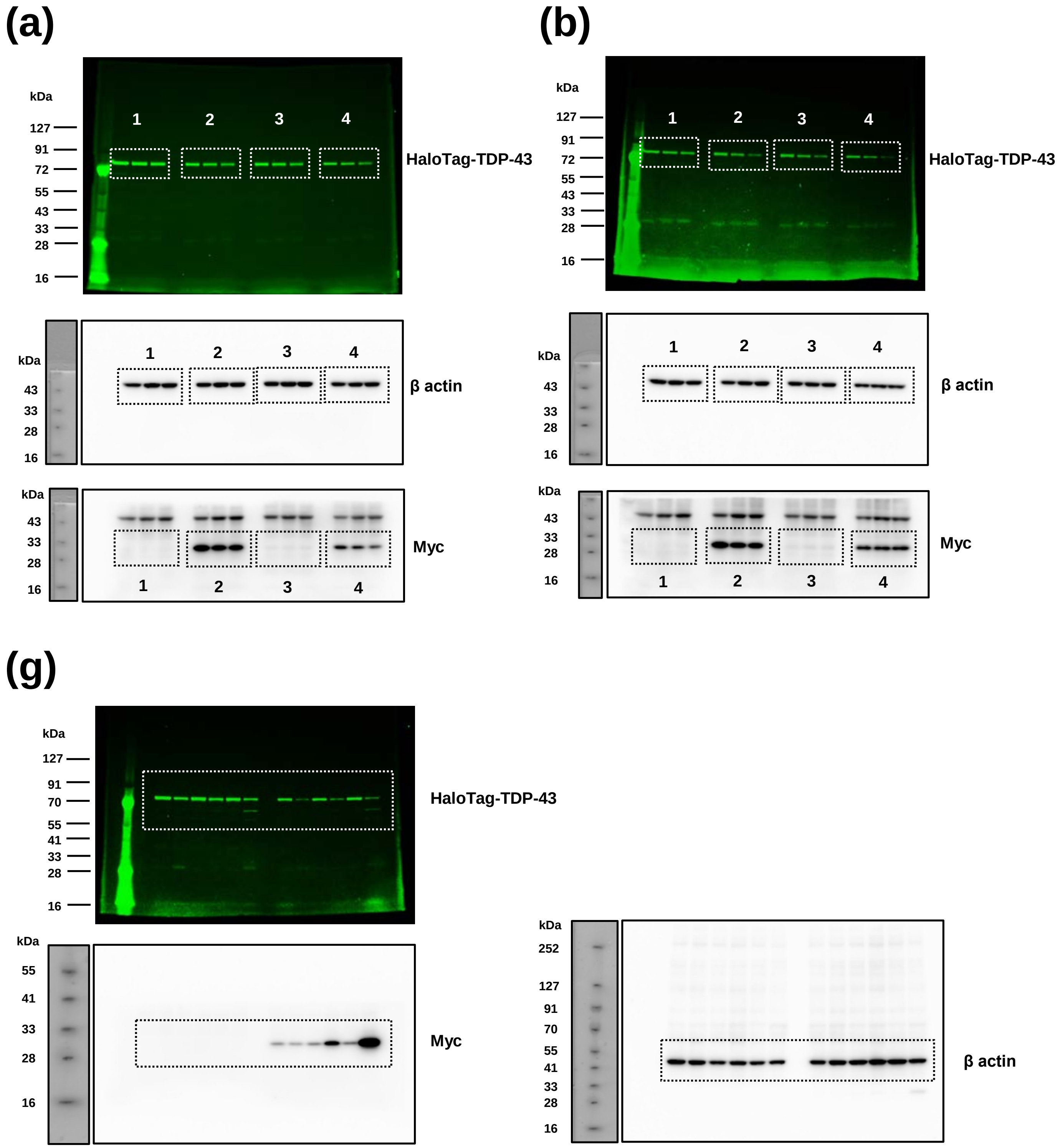

Original Western blots of trimmed panels in Figure 4.
1; vector control, 2; VH_VL, 3; VH_VL-CL1, 4; VH_VL-CMA **(panel a, b)**.

Supplementary Figure S13

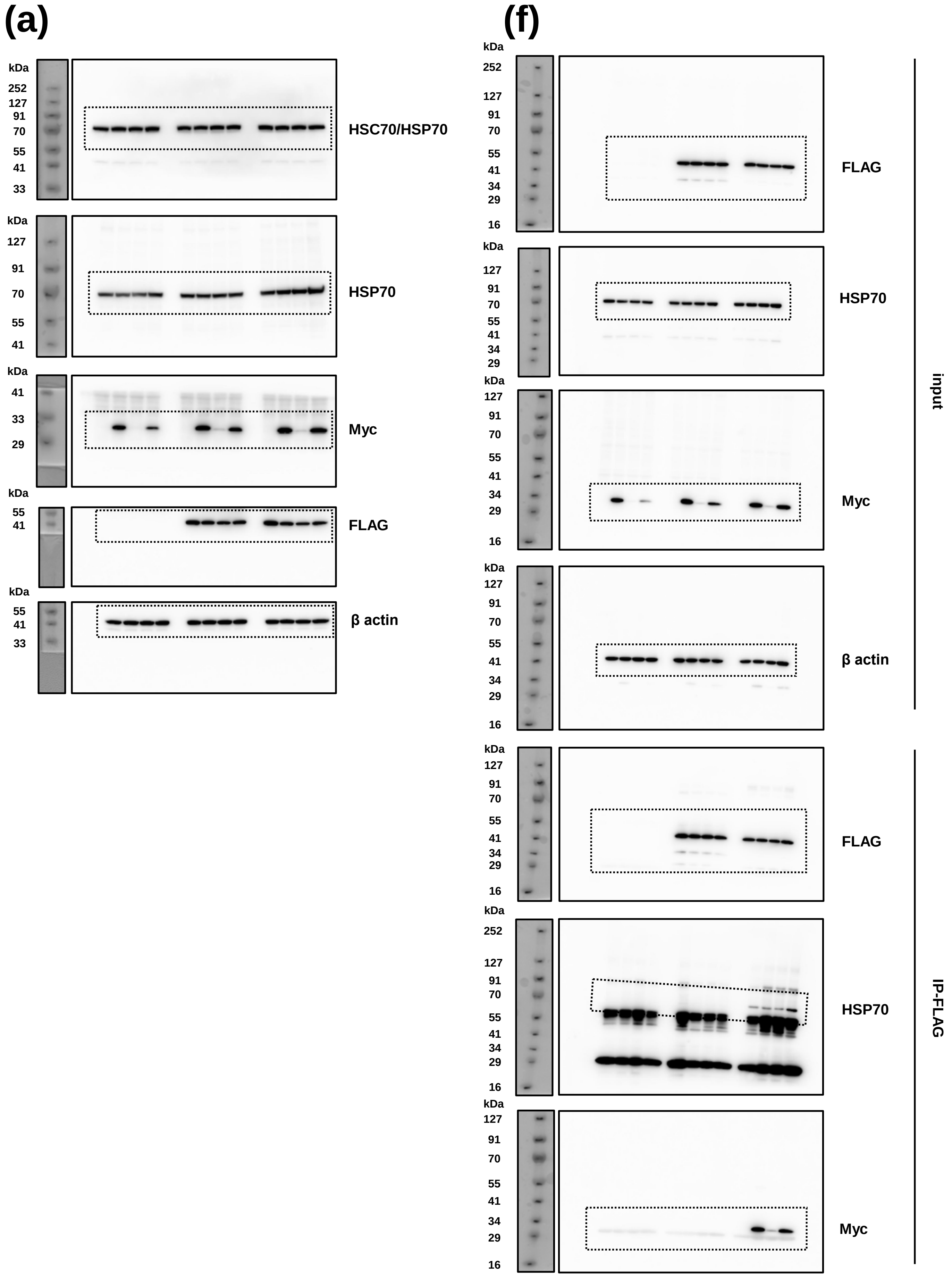

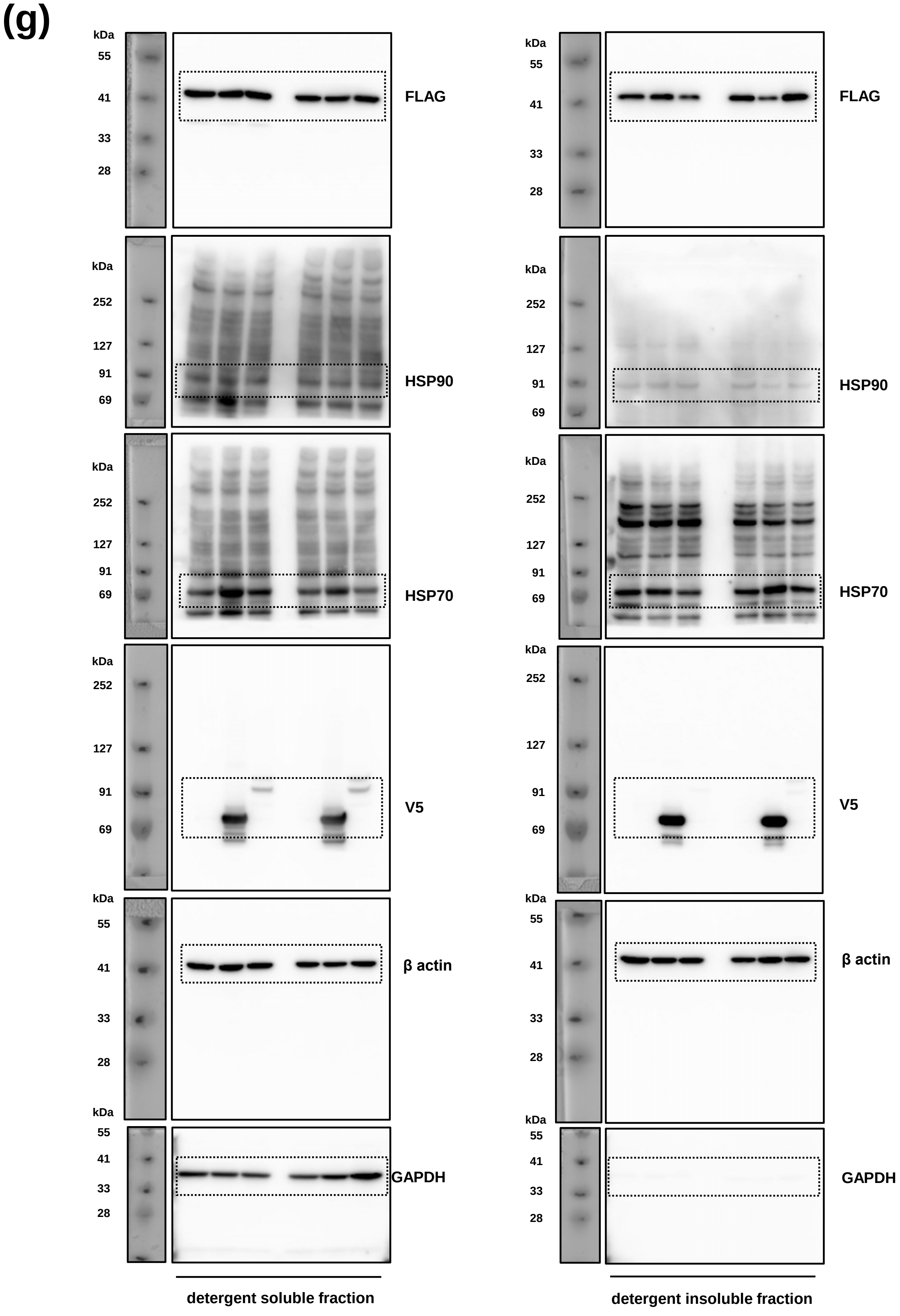

Original Western blots of trimmed panels in Figure 6.

Supplementary Figure S14

(d)

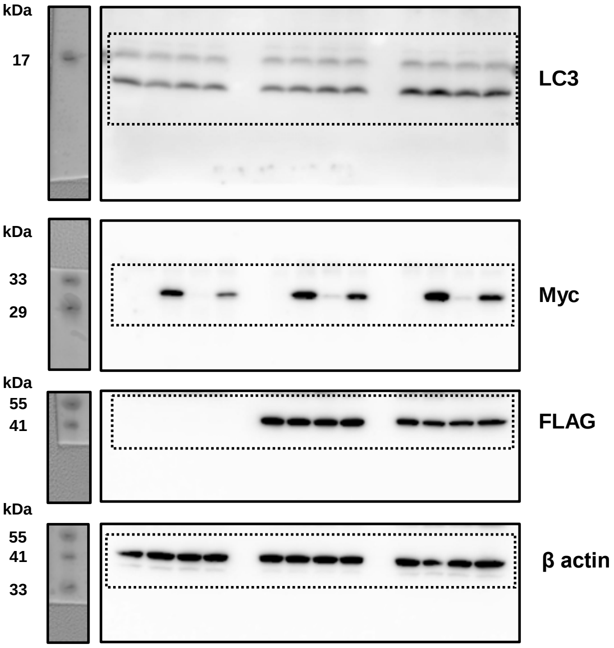

Original Western blots of trimmed panels in Supplementary Figure S6.
